# Interspecies hybridization as a route of accessory chromosome origin in fungal pathogens

**DOI:** 10.1128/mbio.03468-25

**Published:** 2026-02-24

**Authors:** Wagner C. Fagundes, Mareike Möller, Alice Feurtey, Rune Hansen, Janine Haueisen, Fatemeh Salimi, Alireza Alizadeh, Eva H. Stukenbrock

**Affiliations:** 1 Environmental Genomics Group, Max Planck Institute for Evolutionary Biology, Plön & Christian-Albrechts University Kiel, Kiel, Germany; 2 Department of Plant Protection, College of Agriculture and Natural Resources, University of Tehran 48424 , Karaj, Iran; 3 Department of Plant Protection, Faculty of Agriculture, Azarbaijan Shahid Madani University 125619 , Tabriz, Iran; Universidad de Cordoba, Cordoba, Spain

**Keywords:** chromosome structure, transposable elements, wild grasses, transcriptomics, histone methylation

## Abstract

Fungal plant pathogens have dynamic genomic architectures that can contribute to rapid evolution and adaptation to new niches. *Zymoseptoria tritici*, an important fungal pathogen of wheat, has a compartmentalized and rapidly evolving genome. In the genome of the reference isolate *Z. tritici* IPO323, 8 of the 21 chromosomes are accessory. In spite of the profound impact on genome organization, the origin of accessory chromosomes in *Z. tritici* is still poorly understood. By combining a multi-omics approach, we discovered an additional chromosome in *Z. tritici* isolates infecting wild grasses from the genus *Aegilops*, and we use this discovery to study the origin of accessory chromosomes. The newly identified chromosome presents characteristics similar to known accessory chromosomes in *Zymoseptoria* species, including presence-absence variation and enrichment with heterochromatin-associated histone methylation marks (H3K27me3). Interestingly, we found an orthologous chromosome in *Zymoseptoria ardabiliae*, a closely related fungal species also infecting wild grasses. This orthologous chromosome also presents accessory chromosome characteristics but lacks the enrichment of heterochromatin-associated methylation marks. Transcriptomic analyses revealed that the orthologous chromosome in *Z. ardabiliae* harbors active transposable elements (TEs) congruent with lower signatures of host-genome defense mechanisms against TE expansion and spread (quantified as repeat-induced-point mutation signatures). Our findings suggest that the chromosome has been exchanged between *Z. tritici* and *Z. ardabiliae* by introgressive hybridization events, underlining the relevance of hybridization in the evolution of new accessory chromosomes. We speculate that the regulation of TEs has not yet occurred on this new accessory chromosome in *Z. ardabiliae*, contributing to its rapid evolution.

**IMPORTANCE:**

Some species of fungi have accessory chromosomes that show presence-absence variation among individuals in a population. In some species, accessory chromosomes can encode important genes, whereas in others, they have been described as selfish elements. The evolutionary origin of accessory chromosomes is still poorly understood. Here, we characterize a new accessory chromosome in a plant-pathogenic fungus. We demonstrate that this chromosome was recently acquired through interspecific hybridization. Using population genomic data, we also characterize the mechanisms by which accessory chromosomes diverge rapidly. Our study demonstrates how the evolution of accessory chromosomes can be shaped by the interplay between transposable element activity and genome defense mechanisms and illustrates a possible route of chromosome emergence in fungal species.

## INTRODUCTION

Many fungal plant pathogens exhibit remarkable variation in genome architecture, even between closely related species (1). Key observations from comparative genome studies involved the identification of distinct and rapidly evolving compartments in genomes of closely related fungal plant pathogen species, including gene-sparse, repeat-rich regions, AT-rich isochores, clusters of tandem duplicated genes, and accessory or lineage-specific chromosomes (2–14). These distinct genome compartments present different rates of mutation and recombination events and contribute to dynamic genome architectures and rapid evolution of fungal pathogens (1, 7, 15, 16).

Fungal accessory chromosomes are generally small (<2 Mb in length), enriched with transposable elements (TEs), and have a lower gene density and transcriptional activity compared to core chromosomes (15, 16). Moreover, accessory chromosomes were experimentally shown to be lost recurrently during mitotic cell divisions and undergo non-mendelian segregation in the fungus *Zymoseptoria tritici*, resulting in a presence-absence variation (PAV) among individuals of a population (17–19). In *Z. tritici* as well as in other species, accessory chromosomes were also shown to be enriched in heterochromatin-associated histone methylation marks (e.g., H3K27me3 and H3K9me3), correlating with the low transcriptional activity found on these chromosomes (15, 18, 20). In some fungal species, accessory chromosomes encode virulence-related genes that can determine disease outcome and host range of specific fungal species or lineages, as reported for *Leptosphaeria maculans* (21), *Fusarium solani* (22), and *Fusarium oxysporum* f. sp. *lycopersici* (3, 23). Despite the PAV between fungal individuals, accessory chromosomes appear to have been maintained over long evolutionary periods, raising questions on the transmission and maintenance of chromosomes over generations and, more importantly, how they originate in the first place. Few studies have experimentally addressed these questions in different fungal pathogens (3, 24, 25), but the evolutionary mechanisms of accessory chromosome origin remain largely unknown.

*Zymoseptoria tritici*, besides being an important fungal pathogen of wheat, is also an important experimental model in studies of fungal genome evolution (26, 27). Coalescence analyses suggest that the emergence of this wheat pathogen from wild ancestors occurred in the Fertile Crescent region in the Middle East and coincided with the domestication of wheat (26). Population genetics studies using polymorphism data and whole-genome sequencing from worldwide *Z. tritici* populations have indicated high levels of genetic variation within and between *Z. tritici* populations and extensive gene flow from regional to continental scales (27–29). The reference genome of *Z. tritici* is one of the first complete haploid fungal genomes with 21 chromosomes sequenced from telomere to telomere in the isolate IPO323, 8 of them being accessory (30). Accessory chromosomes in *Z. tritici* show hallmarks that distinguish them from the core chromosomes: whole chromosome PAV between isolates; TE richness; low gene density; enrichment of the heterochromatin-associated methylation mark H3K27me3; and low gene transcriptional activity not only *in vitro* but also *in planta* (20, 30–33). In contrast to other fungal pathogens, accessory chromosomes in *Z. tritici* are not enriched in virulence-related genes, and no virulence factors have yet been identified on these chromosomes, even though findings suggest that they can confer a fitness cost in a wheat cultivar-specific manner (34).

The closest known relatives of *Z. tritici,* namely *Zymoseptoria brevis*, *Zymoseptoria pseudotritici,* and *Zymoseptoria ardabiliae,* are found to be associated with different wild grasses in the Middle East, e.g., *Lolium* spp., *Elymus repens*, and *Dactylis glomerata* (35–37). Comparative genomics analyses using long-read assemblies and gene annotations of different *Zymoseptoria* species have revealed a diverse set of genes and a large distribution of accessory chromosomes within and between species, suggesting that genome compartmentalization is an ancestral trait in the *Zymoseptoria* genus (38, 39). Comparative analyses of genome composition across different *Zymoseptoria* species revealed a prominent role of interspecific hybridization in species evolution. Notably, at the center of origin of these pathogens, where they co-exist, hybridization appears to occur readily and shape patterns of genetic variation (36, 40–42). Intriguingly, hybridization can also confer adaptive introgression of functionally relevant traits as demonstrated for the DNA methyltransferase (DNMT) gene *dim2* (41). DNMTs are a class of enzymes involved in DNA methylation in eukaryotes, playing important roles such as conditional expression of genes and the suppression of transposon activity (43–45). In a previous study, Möller and colleagues (41) showed that *Z. tritici* populations differ in the extent of DNA methylation (5mC), a difference that can be attributed to the presence or absence of a functional *DIM2* gene. In some isolates, the *DIM2* gene has been multiplied by the activity of transposable elements, which was followed by mutagenesis through the fungal-specific mechanism repeat-induced point (RIP) mutations that targeted repeated sequences. In isolates with multiple copies, RIP has conferred inactivation of the functional DIM2 gene. Interestingly, *Z. tritici* isolates from the center of origin of the species in the Middle East have “intact” DIM2 copies, likely due to recurrent introgression events with wild grass-infecting isolates that co-exist in the same geographical regions (36, 40, 41). However, to what extent hybridization impacts accessory chromosome evolution has so far not been addressed.

Recently, we have identified host-specific populations of *Z. tritici* infecting wild grass species of the genus *Aegilops*. Preliminary analyses indicate that *Aegilops*-infecting *Z. tritici* isolates are distinct from wheat-infecting *Z. tritici* isolates and from closely related *Zymoseptoria* species (46). These two distinct populations provide a unique model system to study recent evolutionary changes in pathogen species adapting to different hosts and environments. Different footprints of positive selection were detected between host-diverging populations, and demography analyses further suggested a split of the wheat- and *Aegilops*-infecting lineages after 10,000 years ago (46). The collection of closely related lineages of *Z. tritici* provides an excellent model system to address the evolution of genome architecture and accessory chromosome composition in natural fungal populations.

In this study, we explore the genomic variation in *Z. tritici* isolates infecting *Aegilops* species. We present a new high-quality reference genome based on long read-sequencing for the *Aegilops*-infecting *Z. tritici* Zt469 coupled with gene and TE predictions and compare the gene content between this isolate and other *Z. tritici* isolates, as well as between closely related *Zymoseptoria* species. Combining different omics approaches, we identified a unique accessory chromosome in *Aegilops*-infecting *Z. tritici* isolates, which has synteny with another accessory chromosome in the closely related *Z. ardabiliae* species. Analyses of the orthologous chromosomes in the two *Zymoseptoria* species reveal different levels of TE expression and host-genome defense mechanism activity against TEs, besides distinct enrichment of heterochromatin-associated methylation marks. We suggest that this chromosome has been exchanged between *Z. tritici* and *Z. ardabiliae* and that interspecies hybridization combined with TE activity can play a prominent role in the evolution and diversification of new accessory chromosomes.

## MATERIALS AND METHODS

### DNA sequencing and whole-genome assemblies

Reference genomes for *Aegilops*-infecting *Z. tritici* isolate Zt469 and *Z. ardabiliae* isolate Za100 were sequenced using the single-molecule real-time (SMRT) PacBio technology. Zt469 was isolated from *Aegilops* spp. in 2018, and the *Z. ardabiliae* isolate Za100 was isolated in 2011 from *Agropyrum tauri*. High-quality DNA was extracted as described previously (47). Library preparations and sequencing were performed at the Max Planck-Genome-Centre, Cologne, Germany, using a PacBio Sequel II platform. Genomes were assembled *de novo* using the SMRT Analysis software version 5 (Pacific Biosciences) using default and “fungal” parameters as previously described (38). We chose the best genome assemblies for further analyses by comparing the assembly statistics generated by the software Quast (48). We retained assemblies with the lowest number of finished contigs, an approximate total length of 41 Mb, and the highest *N*_50_ value. A previous version of the Zt469 genome assembly based on the long-read sequencing (PacBio) has been published (41), and an improved version of this genome assembly is presented here. In this version, we have filtered the Zt469 raw assembly to match filtering steps done previously and improve the overall assembly quality by removing low-quality contigs (10, 38) (see Table S1 and Text S1). Telomeric repeats (“CCCTAA”) for each contig were identified as previously described using Bowtie2 (38, 49) (see Table S1 and Text S1). Genome assemblies and gene annotations based on long-read sequencing (PacBio) of the *Z. tritici* isolates Zt05 and Zt10 and of the closely related species *Z. brevis* (Zb87), *Z. passerinii* (Zpa63), *Z. pseudotritici* (Zp13), and *Z. ardabiliae* (Za17) were obtained from a previous study (38) (Table S1). Genome assembly and gene and repeat annotations from the reference wheat-infecting *Z. tritici* isolate IPO323 were obtained from other previous studies (30, 39, 50) (Table S1). Population data from *Aegilops*- and wheat-infecting *Z. tritici* and from additional *Z. ardabiliae* isolates were also obtained from other studies (36, 37, 46, 51). Short-read sequencing data from the *Z. passerinii* isolate Zpa796 were obtained from Rojas-Barrera and colleagues (52).

### Repeat and gene annotations

Gene and TE annotations for Zt469 and Za100 genomes were performed using previously published pipelines (38, 50, 53). A more detailed description of the pipelines and tools used is provided in Text S1. In brief, gene annotations were performed using a combination of three different methodologies: one consisting of *ab initio* predictions, and two using RNA-seq data to predict gene coordinates (Text S1). For TE annotations, we used the TEdenovo tool from the REPET package (https://urgi.versailles.inrae.fr/Tools/REPET) (54, 55), following the developer’s recommendations and default parameters. TEs were classified according to the nomenclature defined by Wicker et al. (56). Following the *de novo* identification of TEs, the consensus TE library was further curated with assistance from MCHelper (57) and finally merged with the pangenome of *Z. tritici* and the library of predicted TEs published previously (53). Annotation of TEs using the final curated TE library was performed with RepeatMasker version 4.1.8 (58) (Text S1). For visualization in Circos plots (59), we calculated gene and TE densities in 100 kb non-overlapping windows using the bedtools version 2.26.0 *makewindows* and *coverage* tools (60). Total percentage coverage of TEs per genome and chromosome sequences was calculated using the “bed_coverage” function implemented in the R package “valr” (61).

We used distinct tools to predict the putative gene functions in the newly assembled Zt469 and Za100 genomes. First, we obtained the amino acid sequence of each gene model and used the tool Predector to predict secreted proteins and PFAM domains (62). Predector uses several tools for fungal secretome and effector analyses, including the software SignalP (versions 3, 4, 5, and 6 [63–66]), TargetP (version 2.0 [67]), DeepLoc (68), TMHMM (69, 70), Phobius (71), DeepSig (72), CAZyme finding (with dbCAN [73]), Pfamscan (74), searches against PHI-base (75), Pepstats (74), ApoplastP (76), LOCALIZER (77), Deepredeff (78), and EffectorP (versions 1, 2, and 3 [79–81]). We furthermore used the software eggnog-mapper to provide additional Clusters of Orthologous Groups (COG), Gene Ontology (GO), and Kyoto Encyclopedia of Genes and Genomes (KEGG) annotations (82). We categorized the gene models into “Effector” and “CAZyme” based on the individual tools’ score thresholds recommended by Predector (Table S2). “Small secreted proteins (SSPs)” category also followed the criteria described previously (39) (Table S2). At last, we used Antismash version 6.0 (fungal version) to detect biosynthetic gene clusters (BGCs) in each genome (83) (Table S2). Gene models that did not belong to any of these categories were classified as “Other.” The outputs of all tools and transcription *in vitro* and *in planta* (when applicable) for each gene model, as well as the genes belonging to each category, can be found in Tables S3 to S5.

### Synteny and orthologous gene analyses

We identified orthologous genes between the *Zymoseptoria* genome assemblies using the software PoFF implemented in Proteinortho6 (84, 85) to account for synteny information. Briefly, for each genome analyzed, we first obtained the predicted amino acid sequence of each CDS gene model. We then used these sequences and the corresponding coordinates in Proteinortho6 with the options “-synteny” to identify synteny of genes and “-single” to report singletons (84, 85). The identified orthogroups were used to visualize synteny between genomes using the software Circos, where “links” between assemblies represent synteny of orthologous genes (59).

### ChIP- and RNA-seq data analyses

Preparation and sequencing of RNA- and ChIP-seq samples of *in vitro* growth are described in Text S1. Additional steps and software used to analyze RNA- and ChIP-seq data can also be found in Text S1. For the *in planta* RNA-seq data of the isolate Zt469, we analyzed infection stage-specific transcriptome data generated in a previous study (46). Sequencing adapters and low-quality reads were trimmed from raw paired-end RNA and ChIP-seq reads using Trimmomatic version 0.39 (86) with the following parameters: LEADING:20 TRAILING:20 SLIDINGWINDOW:5:20 MINLEN:50. Low-quality nucleotides (*Q* < 20) in RNA-seq reads were further masked using FASTX-toolkit version 0.0.13 (http://hannonlab.cshl.edu/fastx_toolkit/). For RNA-seq data, quality-trimmed and masked reads were mapped to the Zt469 and Za100 genomes using HISAT2 with distinct settings for gene and TE expression analyses (Text S1), while for ChIP-seq, read mapping was performed using Bowtie2 with default settings (49, 87). Conversion, sorting, merging, and indexing of alignment files were performed using SAMtools version 1.7 (88). PCR duplicates in ChIP-seq reads were removed using the Picard version 2.26.2 MarkDuplicates tool (https://broadinstitute.github.io/picard/). We used the software HOMER (89) to detect methylation-enriched regions in the ChIP mappings (Text S1). We called methylation-enriched peaks individually for each replicate and merged the peaks for each genome and histone methylation mark with bedtools *intersect* version 2.26.0 (60). Only enriched regions found in all replicates were considered for downstream analyses. Sequence coverage of methylation-enriched regions per contigs was calculated using bedtools *genomecov* version 2.26.0 (60). We also calculate the overlap of TEs and genes per methylation mark using bedtools *annotate* version 2.26.0 (60). For RNA-seq *in vitro* and *in planta*, we accessed gene and TE expression as transcript per million (TPM) as previously described (38, 41) (Text S1). For these steps, we used the software htseq-count version 2.0.2 (90) for read counts per gene, DESeq2 (91) for differential gene expression analysis, and the TEtranscript pipeline for TE expression (92, 93) (Text S1). For Circos plot representations (59), we first calculated the mean TPM between replicates and then the averages per 100 kb non-overlapping windows in each unitig based on the log2(TPM + 1) values. For RNA-seq read mapping statistics, we used the tool Qualimap bamQC version 2.2.1 (94).

### Repeat-induced point mutation analyses

We quantified RIP signatures along the Za100 and Zt469 genomes and among TE copies within each genome. To this end, RIP-like signatures and the large RIP-affected genomic regions (LRARs) were calculated using the RIPper software (95). Genome-wide RIP composite indices were calculated using default parameters of 1,000 bp windows with a 500 bp step size and following the formula: (TpA/ApT) – (CpA þ TpG/ApC þ GpT) (95). LRARs were defined as genomic regions (windows) consecutively affected by RIP that are more than 4,000 bp in length. To access the RIP composite index of each TE copy, we calculated the indices in 50 bp nonoverlapping windows using a previously published custom script (50). Regions were considered to be affected by RIP when the composite index is >0 (95). To correlate RIP signatures with nucleotide compositions, we determined the composition of dinucleotides (dimers) and calculated their frequencies in the Zt469 and Za100 genomes and separately for unitig 3 and unitig 9 using the tool “compseq” (“-word 2”) from the EMBOSS suite package (96). Deviation of dinucleotide frequencies was calculated by relating observed frequencies to expected frequencies (assuming equal frequencies of 0.065 for every dinucleotide).

### Karyotype analysis

We compared the genome structure and chromosome presence-absence variation of different *Z. tritici* and *Z. ardabiliae* isolates using two approaches. First, whole-genome sequencing reads were trimmed using Trimmomatic version 0.39 (86) using the following parameters: LEADING:20 TRAILING:20 SLIDINGWINDOW:5:20 MINLEN:50. Trimmed reads were then mapped to the *Z. tritici* reference genome IPO323 (30) as well as to the newly assembled *Aegilops*-infecting *Z. tritici* Zt469 and *Z. ardabiliae* Za100 genomes using bwa-mem version 0.7.17 (97). After mapping, we normalized the coverage of mapped reads by each assembly size using the deepTools version 2.0 *bamCoverage* tool and the options “-of bigwig --binSize 10 --normalizeUsing RPGC --ignoreDuplicates” (98, 99). Normalized alignment files were then visualized with the Integrate Genome Viewer software version 2.8.2 (100). Only the Zt469 and Za100 contigs containing telomeric repeats and/or larger than 100 kb were kept for visualization. A second approach involved the analysis of chromosome PAV by pulsed field gel electrophoresis (PFGE) and Southern blot techniques (101). Preparation of non-protoplast plugs for PFGE analyses was done as described previously (19). As a comparison to Zt469, we also prepared non-protoplast plugs for PFGE analyses for the reference *Z. tritici* IPO323 isolate (with our lab ID Zt244) (30), the wheat-infecting *Z. tritici* isolate Zt10 (strain Zt366 [26]), and the *Aegilops*-infecting *Z. tritici* isolate Zt501 (46). PFGE running conditions and Southern blot analyses were done as previously described (19, 101) and are further summarized in Text S1.

### Introgression and nucleotide diversity analyses

We calculated Patterson’s *D*-score, also known as the ABBA-BABA test (102, 103), to determine the occurrence of interspecific gene flow between *Z. tritici* and *Z. ardabiliae*. The test is based on a resolved phylogeny among four taxa (P1, P2, P3, and O) and determines, along the genome, the proportion of derived (“B”) and ancestral (“A”) alleles as defined by the outgroup (O). An excess of shared derived alleles indicates gene flow between two of the taxa (102, 103). In our tests, we placed either the *Aegilops*-infecting *Z. tritici* isolate Zt469 or the *Aegilops*-infecting *Z. tritici* isolate Zt436 in position P1, the wheat-infecting *Z. tritici* isolate Zt565 or the wheat-infecting isolate Zt668 in position P2, and either the *Z. ardabiliae* isolate Za100 or Za98 in position P3 (Fig. S1). We performed the tests with two different *Aegilops*-infecting *Z. tritici* isolates and two different *Z. ardabiliae* isolates to check if gene flow would occur only in isolates carrying the unitig 9 (in the case of Zt469) or unitig 3 (in the case of Za100). For all tests, we used the *Z. passerini* Zpa796 isolate as the outgroup (O). A negative *D* statistic in these configurations is indicative of gene flow between *Aegilops*-infecting *Z. tritici* (P1) and *Z. ardabiliae* (P3), while positive scores indicate gene flow between wheat-infecting *Z. tritici* (P2) and *Z. ardabiliae* (P3). Details of genome data filtering and processing for ABBA-BABA tests are provided in Text S1.

For the nucleotide diversity analyses in the syntenic unitig 9 and unitig 3 chromosomes, we used single nucleotide polymorphisms (SNPs) obtained from population data of *Aegilops*-infecting *Z. tritici* and *Z. ardabiliae* isolates (Table S6). The complete description of whole-genome sequencing read trimming, mapping, and variant calling is provided in Text S1. From the resulting VCF files, we calculated nucleotide diversity (π) (104) in 1 kb windows using VCFtools version 0.1.13 (105) with the haploid mode fork provided by Julien Y. Dutheil (https://github.com/vcftools/vcftools/pull/69). We compared nucleotide diversity between unitig 9 in Zt469 and unitig 3 in Za100 using polymorphism data from three *Aegilops*-infecting *Z. tritici* and the three *Z. ardabiliae* isolates that contained these chromosomes as identified by the read-mapping approach. The three *Aegilops*-infecting *Z. tritici* isolates were selected based on the lowest pairwise identity-by-state similarity values acquired using PLINK version 1.07 (106) and the generated VCF file in order to keep the most genotypically diverse isolates.

## RESULTS

### A new accessory chromosome in *Aegilops*-infecting *Z. tritici*

**Fig 1:**
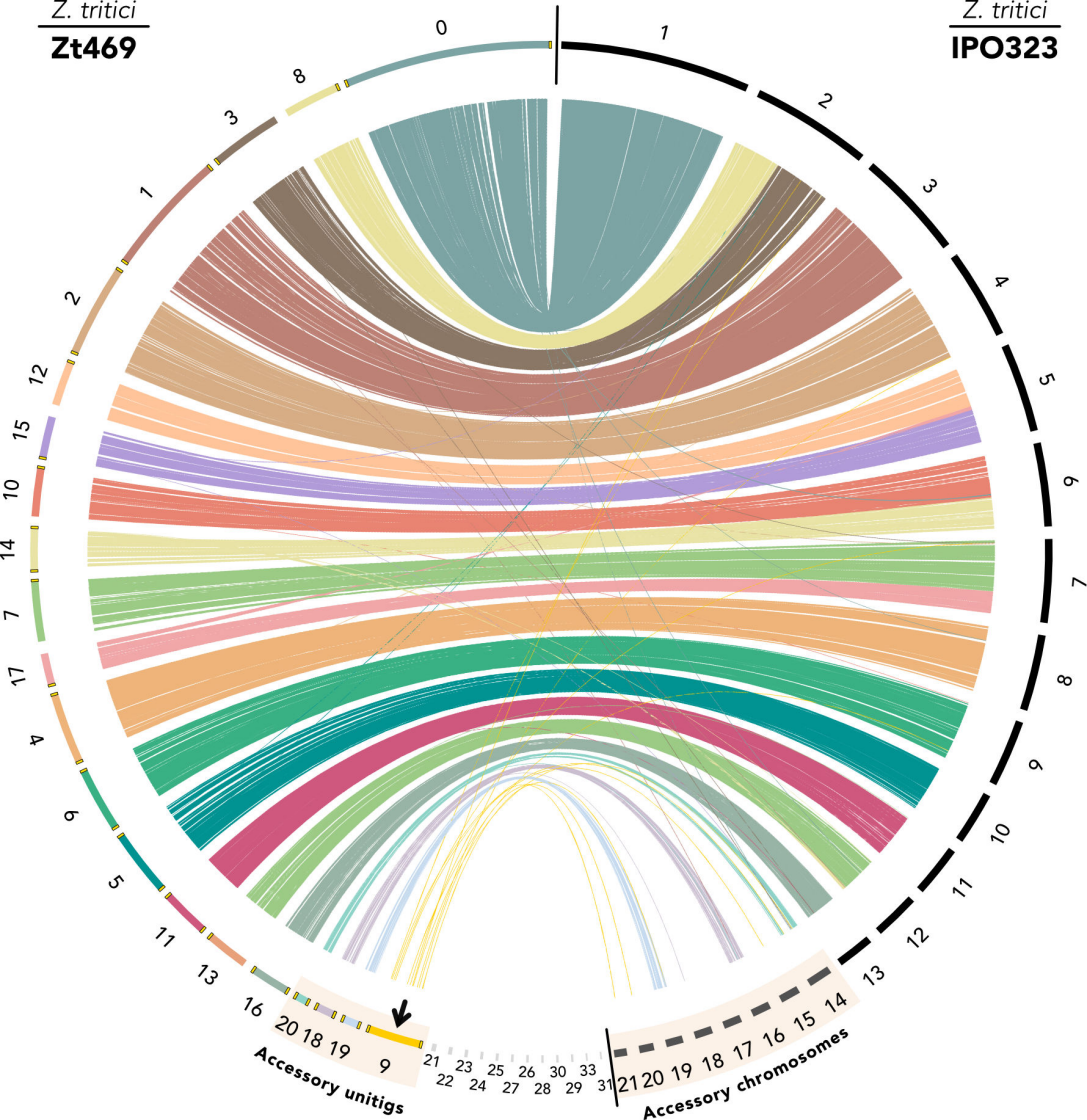
Genome synteny between *Z. tritici* isolates Zt469 and IPO323. Synteny analysis was performed between the reference wheat-infecting *Z. tritici* isolate IPO323 (right) and the *Aegilops*-infecting *Z. tritici* isolate Zt469 (left) based on the predicted gene models. Each color represents a different unitig in Zt469, and colored lines (“links”) represent homologous genes. Small unitigs (>50 and < 300 kb in length) are shown in light gray, and telomeric repeats are indicated in yellow. A new chromosome, referred to as “unitig 9,” is identified in Zt469 and shows no synteny with any particular portion of the IPO323 genome (black arrow). Unitigs in Zt469 are ordered following the synteny with the reference IPO323 genome. Accessory chromosomes and unitigs on the IPO323 and Zt469 genomes are highlighted.

We first used high-quality genome data from *Z. tritici* isolates that infect *Aegilops* spp. or wheat to characterize the extent of structural conservation between the two host-specific lineages. In this study, we improved the genome assembly of *Aegilops*-infecting *Z. tritici* isolate Zt469 reported previously (41) by removing low-quality contigs from the raw assembly (see Materials and Methods and Text S1). These filtering steps resulted in a final assembly consisting of 45 contigs, 13 of which contain telomeric repeats at both ends and most likely represent whole chromosome assemblies (Table S1 and Text S1). Analyses of homologous genes between the reference wheat-infecting *Z. tritici* isolate IPO323 (30) and the *Aegilops*-infecting *Z. tritici* isolate Zt469 revealed a high extent of synteny between the 13 core chromosomes described for IPO323, whereas only 3 out of 8 accessory chromosomes were found to have synteny with chromosomes of Zt469 (unitigs 18, 19, and 20) (Fig. 1). Unexpectedly, this analysis also revealed a complete chromosome (with telomeric repeats on both ends) in Zt469 with no alignments to any specific chromosome of the reference strain IPO323, namely “unitig 9” (Fig. 1). Only 4.3% (13/299) of the predicted genes on unitig 9 have homologs in the IPO323 genome, and the 13 genes are distributed across different chromosomes of IPO323 (Fig. 1).

To further validate the correctness of our genome assembly and the presence and size of unitig 9 in Zt469, we conducted PFGE followed by Southern blot analysis, which confirmed the presence of a chromosome in the expected size (~1.6 Mb) in Zt469 and the absence of the chromosome in the *Z. tritici* IPO323 isolate and in the other two randomly selected *Z. tritici* isolates analyzed (Fig. S2).

We next asked if unitig 9 could be present in other wheat-infecting *Z. tritici* isolates. To this end, we took advantage of the 1,000-genome data set (29) representing *Z. tritici* populations collected from different wheat-growing areas on different continents. Based on sequence homology to the *de novo* genome assemblies of the 1,000 *Z. tritici* isolates (Text S1), we did not find hits with long stretches of similarity to unitig 9, suggesting that the accessory chromosome indeed is unique to the Iranian wild grass-infecting *Z. tritici* (Fig. S3 and Table S7).

### Unitig 9 exhibits presence-absence variation in *Aegilops*-infecting *Z. tritici* isolates

**Fig 2:**
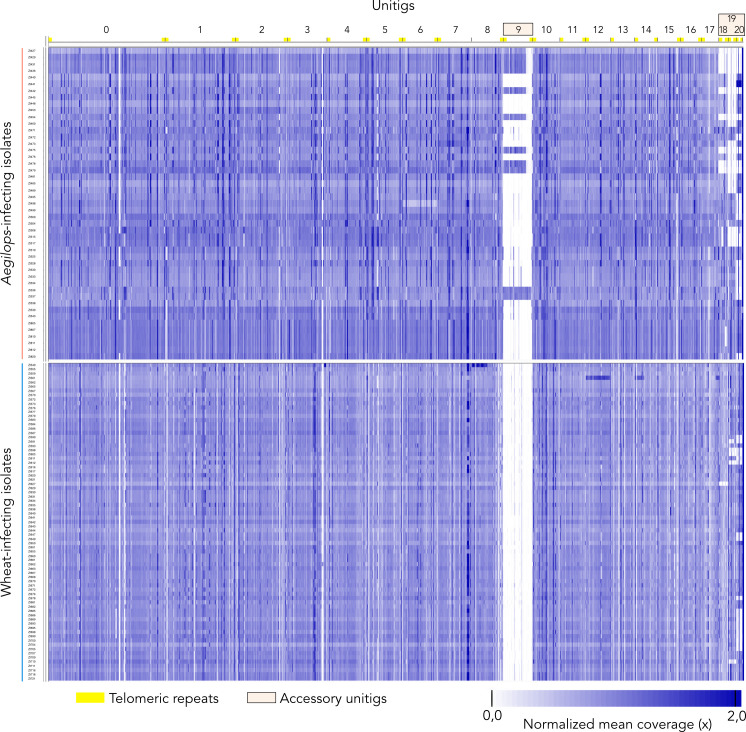
Unitig 9 shows presence-absence variation in *Aegilops*-infecting *Z. tritici*. Chromosome presence-absence variation in host-diverging *Z. tritici* populations was analyzed by read mapping to the *Z. tritici* Zt469 genome assembly. The heatmap represents the normalized mean coverage of reads mapped to each position of the genome assembly in bins of 10 bp. Except for unitig 9, only unitigs syntenic to the *Z. tritici* IPO323 reference genome are shown and sorted in descending order of length. Darker colors represent regions of higher coverage, e.g., repetitive elements or duplications. Accessory unitigs and telomeric repeats are indicated.

In order to examine the presence of unitig 9 in other sympatric *Z. tritici* isolates collected in the Middle East, we conducted genome-wide comparative analyses to identify chromosome presence-absence variation between Iranian *Z. tritici* isolates infecting leaves of wheat and *Aegilops* spp. collected in agricultural fields and natural grasslands, respectively. First, using the IPO323 reference genome and a normalized read coverage approach, we found extensive PAV of the accessory chromosomes for all analyzed *Z. tritici* isolates (Fig. S4). Hereby, we also find that the wheat-infecting isolates exhibited an overall higher presence of these chromosomes when compared to the *Aegilops*-infecting ones (Fig. S4). Next, we used the same mapping approach based on the *Aegilops*-infecting Zt469 reference genome. Hereby, we observe a similar pattern of chromosome PAV for the three shared accessory unitigs (unitigs 18, 19, and 20), while unitig 9 was completely absent in all wheat-infecting isolates (Fig. 2). When analyzing the *Aegilops*-infecting population, we observe, however, that unitig 9 was present in 11 out of 47 isolates, while the accessory unitigs 18, 19, and 20 occurred more frequently in the population (among the 47 isolates in 38, 24, and 23 isolates, respectively) (Fig. 2). While we focused on the presence-absence variation of accessory chromosomes, we note that the coverage maps also reveal putative duplicated chromosome fragments and aneuploidies of duplicated accessory chromosomes. These are visible as darker blue regions in the coverage plots (Fig. 2; Fig. S4). Taken together, these results indicate that unitig 9 is a chromosome present only in *Aegilops*-infecting *Z. tritici* populations.

### Unitig 9 exhibits accessory chromosome hallmarks

**Fig 3:**
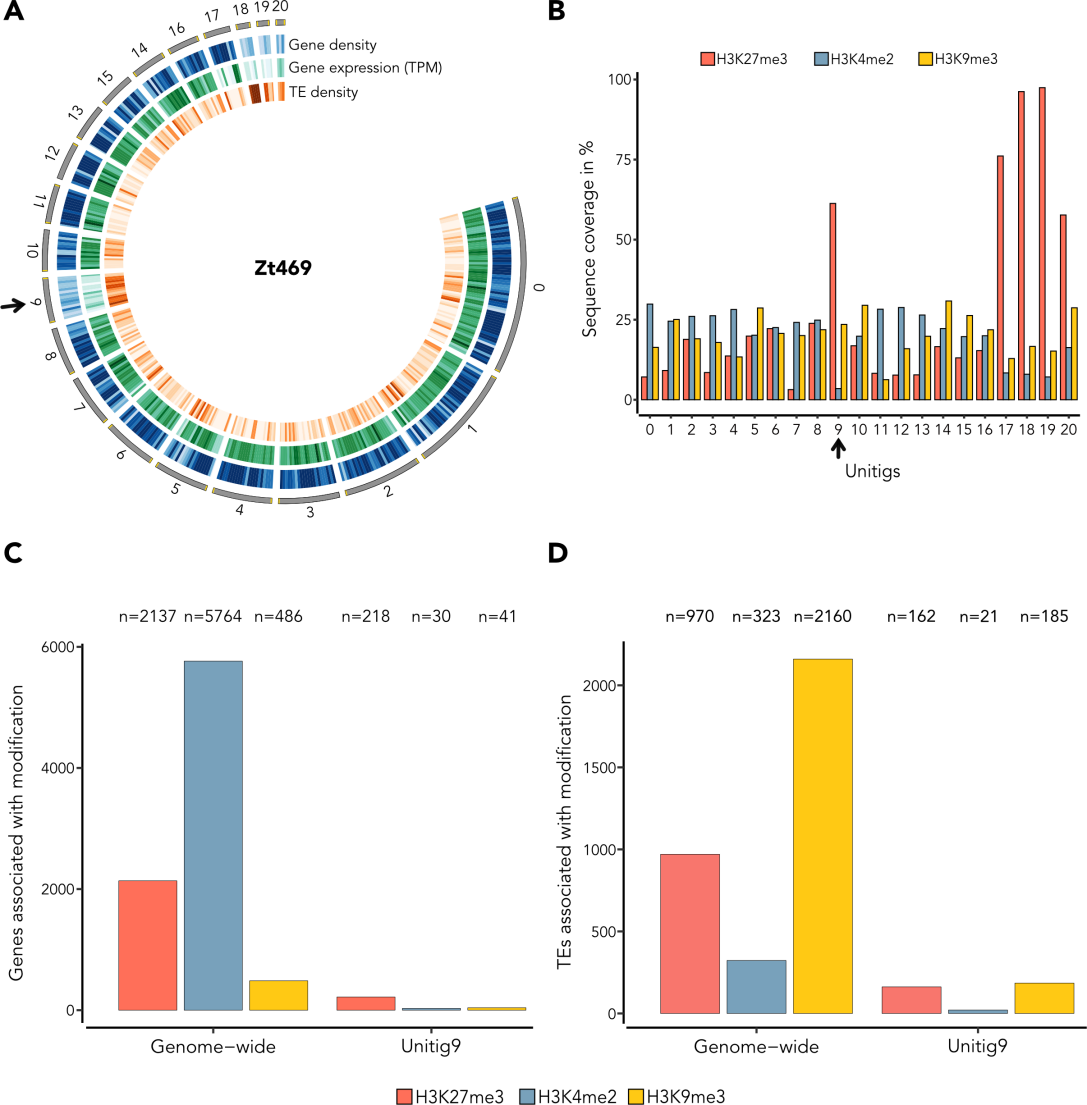
Unitig 9 presents accessory chromosome characteristics. (**A**) Circos plot representing different features along 20 Zt469 unitigs. Unitigs are represented by the dark gray segments sorted by length. Telomeric repeats are indicated in yellow. Tracks from outside to the inside are heatmaps in 100 kb windows representing, respectively, gene density (blue), gene expression *in vitro* in log2(TPM + 1) (green), and TE density (orange). Darker heatmap colors indicate higher values. Unitig 9 is indicated by a black arrow. (**B**) Bar plot displaying the percentage of sequence coverage of each Zt469 unitig with the heterochromatin methylations H3K27me3 (red bars) and H3K9me3 (yellow bars), and the euchromatin methylation H3K4me2 (blue bars) marks relative to unitig length. Unitig 9 is indicated by a black arrow. In both panels, except for unitig 9, only unitigs syntenic to the *Z. tritici* IPO323 reference genome are shown. (**C and D**) Bar plots showing the number (*N*) of genes (**C**) and TEs (**D**) associated with each methylation mark (H3K4me3, H3K9me3, and H3K27me3) genome-wide or in unitig 9 specifically.

Considering the PAV of unitig 9 among *Aegilops*-infecting *Z. tritici* isolates, we hypothesized that unitig 9 represents a new accessory chromosome. We, therefore, set out to investigate if unitig 9 shares the same hallmarks described for accessory chromosomes in *Zymoseptoria* species, e.g., low gene density, high TE density, and low transcriptional activity (1, 30, 38). Gene and TE annotations, as well as transcriptome analyses *in vitro,* revealed that unitig 9 has a lower gene density (average of 0.39 genes/kb), higher TE density (average of 0.35 TEs/kb), and lower transcriptional activity when compared to core unitigs of similar size (e.g., unitig 8; 0.83 genes/kb and 0.31 TEs/kb on average) (Fig. 3A), consistent with known signatures of other accessory chromosomes in *Z. tritici* (30, 33, 38). The smaller unitigs 18, 19, and 20, which we also consider as accessory chromosomes, exhibited similar characteristics (Fig. 3A).

In line with these results, *in vitro* chromatin immunoprecipitation in combination with high-throughput sequencing (ChIP-seq) targeting three histone methylation marks (H3K4me2 for euchromatin and H3K27me3 and H3K9me3 for facultative and constitutive heterochromatin, respectively) also revealed that unitig 9, together with unitigs 18, 19, and 20, is enriched with the H3K27me3 mark (Fig. 3B; Table S8). We also observed that 72.9% of genes annotated in unitig 9 (218/299) are associated with H3K27me3, corroborating the low transcriptional activity observed for these genomic regions (Fig. 3C). At both genome-wide and unitig 9 levels, TEs are more associated with the constitutive heterochromatin mark H3K9me3 (Fig. 3D), similar to what was observed in previous studies (20, 107). We also found the typical signatures of accessory chromosomes on unitig 17 (Fig. 3A and B; Table S8). Interestingly, this unitig is syntenic with the right chromosome arm of chromosome 7 in the *Z. tritici* IPO323 reference genome (Fig. 1). It has been previously proposed that chromosome 7 in IPO323 may represent a fusion event of an accessory chromosome to a core chromosome (20), and our findings add further support to this hypothesis. Altogether, our results suggest that the novel unitig 9, along with unitigs 18, 19, and 20, represents accessory chromosomes in *Aegilops*-infecting *Z. tritici* isolates and carries similar hallmarks to the known accessory chromosomes in other *Zymoseptoria* species.

### High proportion of unitig 9 genes have orthologs in the sister species *Z. ardabiliae*

Based on the previous finding that only a few genes in unitig 9 have homologs in the reference *Z. tritici* IPO323 isolate, we further inspected the orthology of unitig 9 genes in other *Z. tritici* isolates and *Zymoseptoria* species. We used previously published and annotated whole-genome assemblies based on long-read sequencing (PacBio) of the *Z. tritici* isolates Zt05 and Zt10 and the closely related species *Z. brevis* (Zb87), *Z. passerinii* (Zpa63), *Z. pseudotritici* (Zp13), and *Z. ardabiliae* (Za17) (38). Moreover, we included a new long-read, high-quality genome assembly of the *Z. ardabiliae* isolate Za100 (Table S1). At the genome-wide level, we identified 7,703 orthologous genes (i.e., orthogroups) distributed among all nine *Zymoseptoria* genome assemblies analyzed (Fig. S5A). Within species, we detected 146 species-specific orthogroups among *Z. tritici* isolates in comparison to 429 species-specific orthogroups shared between the two *Z. ardabiliae* isolates analyzed (Fig. S5A). Surprisingly, we found that a large number of orthogroups were specifically shared between Zt469 and Za100 (141 orthogroups), just a few orthogroups less than the number of orthogroups shared between all *Z. tritici* genomes (146 orthogroups) (Fig. S5A). In fact, comparisons between the *Z. tritici* isolates IPO323 and Zt469 and two *Z. ardabiliae* isolates (Za17 and Za100) revealed a considerably larger number of orthogroups shared between the *Aegilops*-infecting *Z. tritici* isolate Zt469 and the *Z. ardabiliae* isolates (number of shared orthogroups: Za17: 59 and Za100: 240) when compared to the intersection between IPO323 and the same *Z. ardabiliae* isolates (number of shared orthogroups: Za17: 37 and Za100: 82; Fig. S5B). The higher number of shared orthogroups distributed genome-wide between the *Aegilops*-infecting *Z. tritici* and *Z. ardabiliae* suggests that interspecific introgression events likely take place between these two species infecting wild grasses.

**Fig 4:**
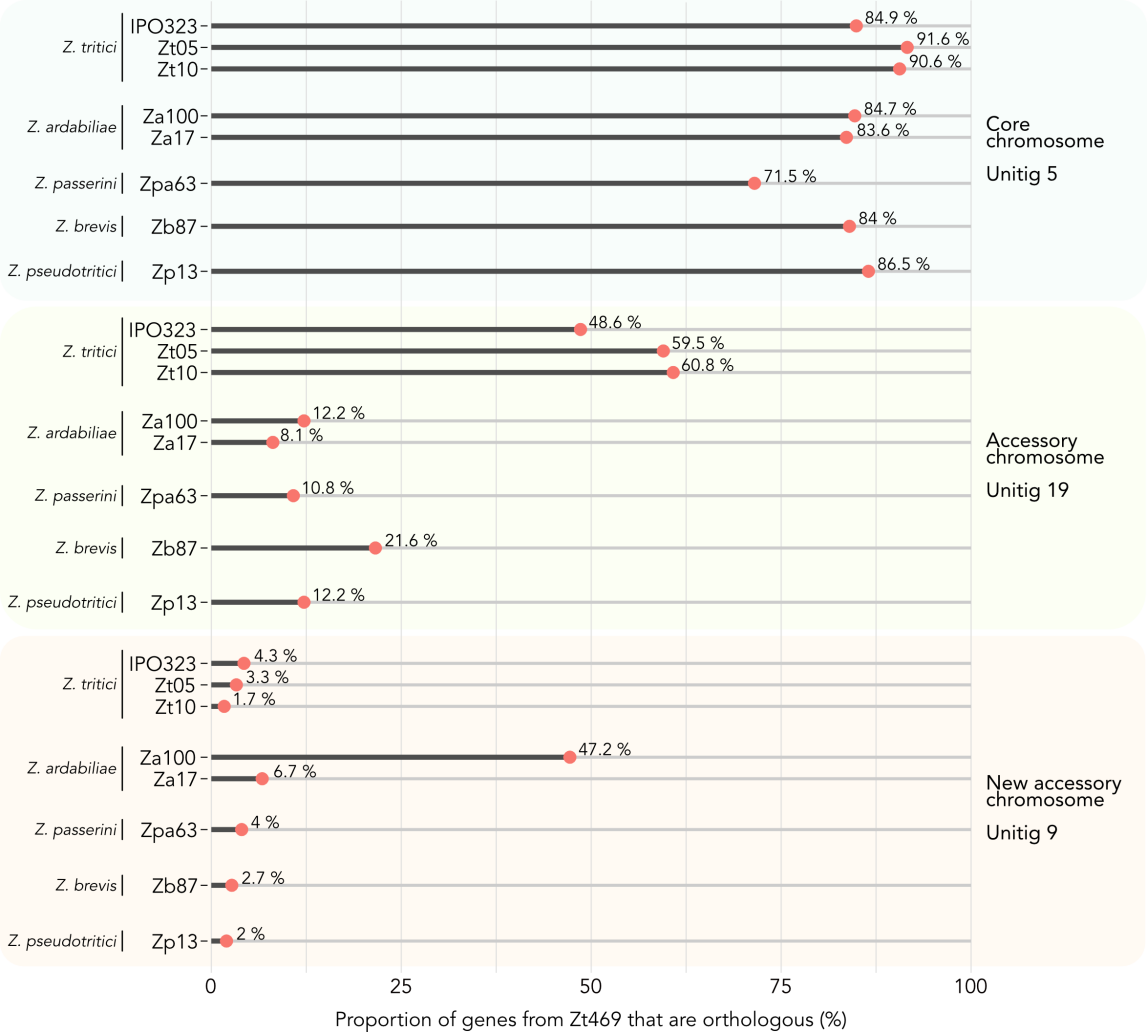
Proportion of Zt469 genes present in other *Zymoseptoria* species genomes. The proportion of genes present in unitig 5 (core), unitig 19 (accessory), and the newly described accessory unitig 9 of Zt469 and shared with other *Zymoseptoria* isolates was calculated for each genome individually. Only orthogroups solely shared between each unitig and the respective isolate genome are considered.

We next focused specifically on the genes on unitig 9 in Zt469 and investigated their proportion in other *Zymoseptoria* genomes. As a comparison, we also analyzed the proportion of genes present in another accessory (unitig 19) and core chromosome (unitig 5) of Zt469, which are shared with the same *Zymoseptoria* isolates. As mentioned above, few orthogroups were observed between unitig 9 of Zt469 and the reference *Z. tritici* isolate IPO323 (only 4.3% orthogroups from unitig 9 were also present in the IPO323 genome) (Fig. 1 and 4). Similarly, we observed that the proportion of unitig 9 genes present in other *Z. tritici* genomes and in genomes of other closely related *Zymoseptoria* species was low, ranging from 1.7% in Zt10 (*Z. tritici*) to 4% in Zpa63 (*Z. passerinii*) (Fig. 4). Unexpectedly, however, we observed a higher proportion of unitig 9 genes with orthologs in the *Z. ardabiliae* isolates Za17 and Za100. Almost half of the genes in unitig 9 had orthologs in Za100 (47.2%), while Za17 had a lower, but still relatively higher compared to the other genomes, proportion of orthologous genes (6.7%) (Fig. 4). This higher proportion of shared orthologs between unitig 9 of Zt469 and the Za100 isolate led us to the hypothesis that unitig 9 may have been exchanged between *Z. tritici* and *Z. ardabiliae* by introgression.

### An accessory chromosome of Za100 is syntenic with unitig 9 in Zt469

**Fig 5:**
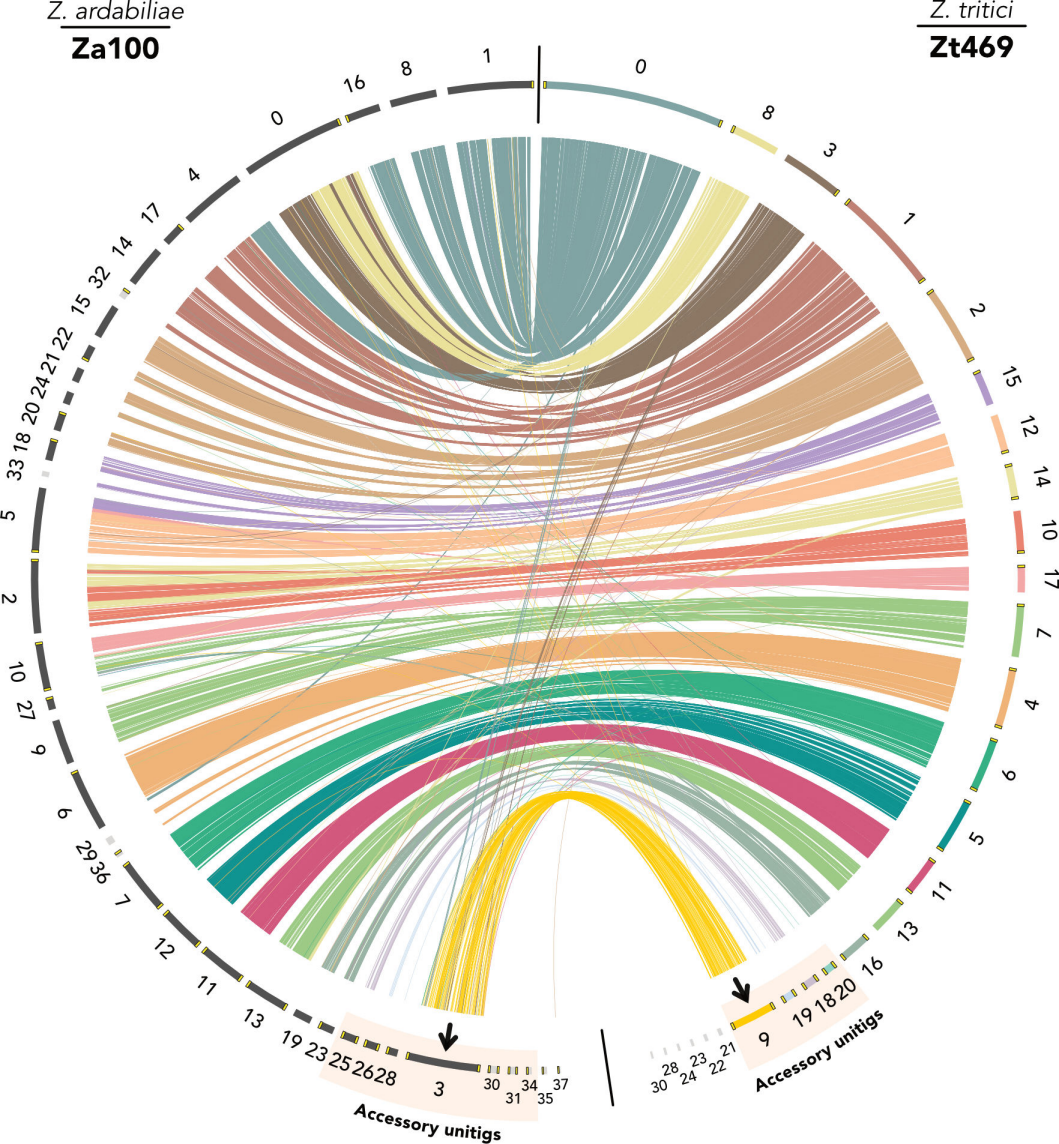
Unitig 3 in Za100 is syntenic to unitig 9 in Zt469. Synteny analysis was performed between the genomes of the *Aegilops*-infecting *Z. tritici* isolate Zt469 (right) and the *Z. ardabiliae* isolate Za100 (left) based on the predicted gene models. Each color represents a different unitig in Zt469. Colored lines (“connections”) represent orthologous genes. Small contigs (>50 and <300 kb in length) are shown in light gray, and telomeric repeats are indicated in yellow. Unitig 3 in Za100 shows synteny with unitig 9 in Zt469 (black arrows). Unitigs in Zt469 are ordered following the synteny with the reference IPO323 genome (Fig. 1). Accessory unitigs on Za100 and Zt469 genomes are highlighted.

The observation that almost half of the genes in unitig 9 had orthologs in Za100 prompted us to further explore the distribution of orthologous genes in the *Z. ardabiliae* genome. As a first step, we analyzed chromosomal synteny between Za100 and Zt469 using the PacBio long-read assemblies and gene annotations. To this end, we plotted the synteny of orthologous genes between the two assemblies using a Circos plot (59). In contrast to the comparison between the *Z. tritici* isolates Zt469 and IPO323 (Fig. 1), we found that unitig 9, to a large extent, is syntenic to unitig 3 in Za100, a presumably completely assembled chromosome comprising telomeric repeats in both ends (Fig. 5). Intriguingly, the *Z. ardabiliae* chromosome is significantly larger than unitig 9 in length (~2.6 Mb compared to ~1.6 Mb). Despite the size difference, we found that 95% (134/141) of the genes of unitig 9 previously observed to have orthologs in Za100 (Fig. 4) localize on unitig 3.

To further characterize the occurrence of unitig 3 in other *Z. ardabiliae* isolates, we determined the presence of unitig 3 in an additional 15 previously published *Z. ardabiliae* genomes collected across different years and locations in Iran (36, 37, 51) (Table S6). Using the PacBio Za100 assembly as reference and a normalized read coverage approach, we found that unitig 3 is present in only three additional isolates in our collection (Za19, Za20, and Za101) (Fig. S6). Similar results were observed when the same *Z. ardabiliae* isolates were mapped to the PacBio Zt469 genome assembly; hereby, only these four isolates (including Za100), as expected, showed the presence of a genomic fragment similar to unitig 9 as reflected by reads mapping to this unitig (Fig. S7). The PAV of unitig 3 among *Z. ardabiliae* isolates collected in different years (2004 and 2011) and locations led us to the conclusion that unitig 3 represents an accessory chromosome at low frequency in *Z. ardabiliae*.

### Unitig 3 in *Z. ardabiliae* has accessory chromosome hallmarks but is not enriched with the H3K27me3 methylation mark

**Fig 6:**
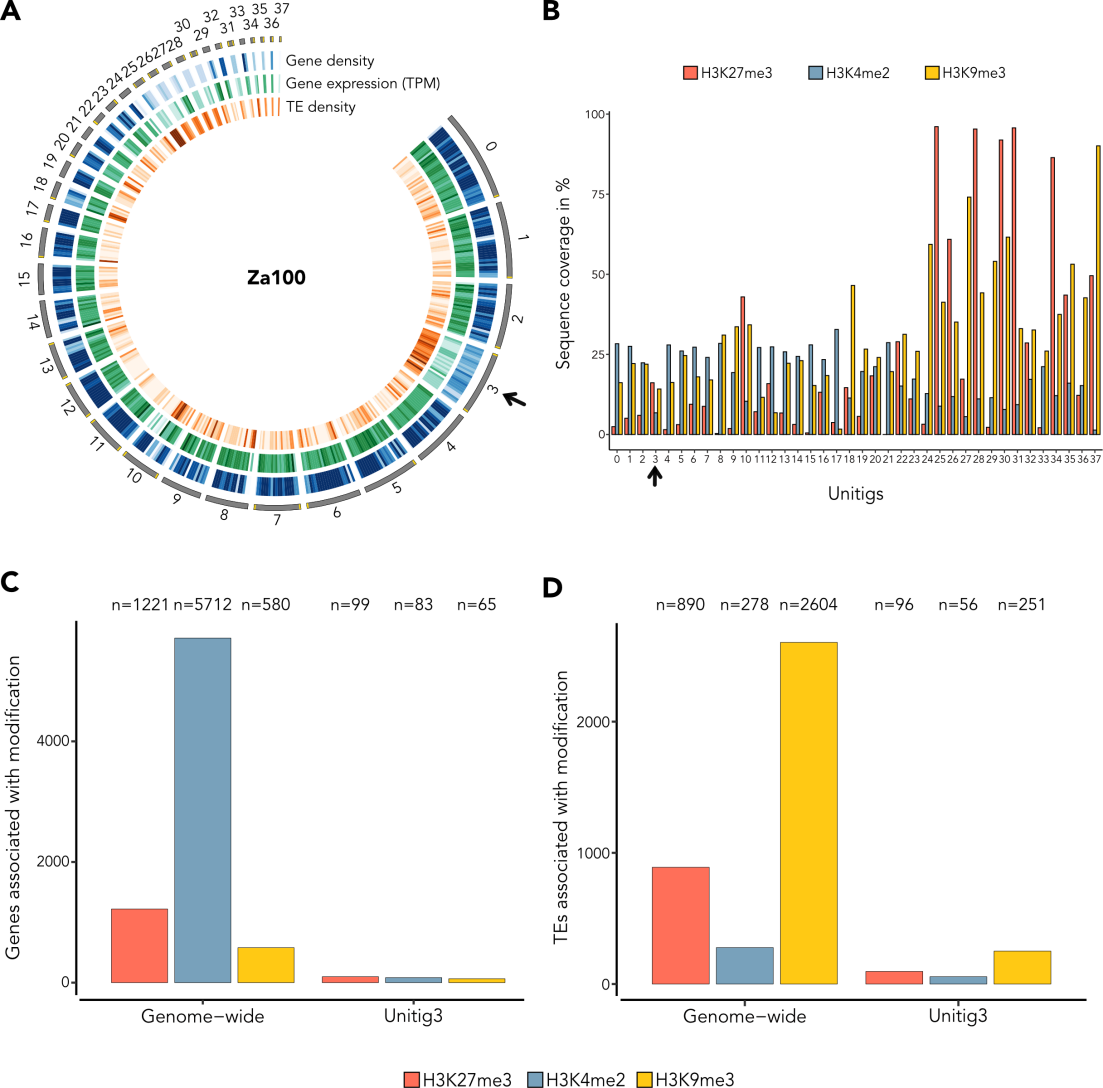
Unitig 3 shows intermediate accessory chromosome hallmarks. (**A**) Circos plot representing different features along the 38 Za100 unitigs. Unitigs are represented by the dark gray segments sorted by length. Telomeric repeats are indicated in yellow. Tracks from outside to the inside are heatmaps in 100 kb windows representing, respectively, gene density (blue), gene expression *in vitro* in log2(TPM + 1) (green), and TE density (orange). Darker heatmap colors indicate higher values. Unitig 3 is indicated by a black arrow. (**B**) Bar plot displaying the percentage of sequence coverage of each Za100 unitig with the heterochromatin methylations H3K27me3 (red bars) and H3K9me3 (yellow bars), and the euchromatin methylation H3K4me2 (blue bars) marks relative to unitig length. Unitig 3 is indicated by a black arrow. (**C and D**) Bar plots showing the number (*N*) of genes (**C**) and TEs (**D**) associated with each methylation mark (H3K4me3, H3K9me3, and H3K27me3) genome-wide or in unitig 3 specifically.

In order to better characterize unitig 3 in Za100, we analyzed the genomic landscape of this chromosome. Similar to unitig 9 in Zt469, we observed a low gene density (average of 0.44 genes/kb), high TE density (average of 0.38 TEs/kb), and low transcriptional activity when compared to unitigs of core chromosomes and of similar size (e.g., unitig 4; 0.86 genes/kb and 0.22 TEs/kb on average) (Fig. 6A). These signatures are consistent with other accessory chromosomes in *Zymoseptoria* species (30, 33, 38). In our search for accessory chromosome signatures, we also identified several other accessory unitigs represented by six shorter unitigs (unitigs 25, 26, 28, 30, 31, and 34) with telomeric repeats in both ends in four of them (Fig. 6A). Intriguingly, we observed that these accessory unitigs do not all comprise the same chromatin patterns, particularly unitig 3. ChIP-seq results targeting three histone methylation marks (H3K4me2, H3K27me3, and H3K9me3) revealed that unitig 3 is not enriched in the heterochromatin methylation mark H3K27me3 when compared to the other accessory unitigs (Fig. 6B; Table S8). Only around 19% (99/519) of genes present in unitig 3 are associated with H3K27me3, while TEs are more associated with H3K9me3 in this unitig (Fig. 6C and D). We also observed accessory chromosome signatures on unitig 10, which is syntenic to unitig 17 in Zt469 (Fig. 5) and to the “accessory arm” of chromosome 7 in *Z. tritici* IPO323 (Fig. 1 [20]). The low enrichment of H3K27me3 in unitig 3 suggests that, even though the unitig has some accessory chromosome hallmarks (high TE, low gene density, and low transcriptional activity), it still has a different profile of histone modifications compared to other accessory chromosomes described in *Zymoseptoria* species.

### Unitig 3 and unitig 9 show high transcription activity of TEs and different genome defense signatures

Considering the differences observed in length and histone methylation marks between unitig 3 in Za100 and unitig 9 in Zt469, we focused on further characterizing the TE landscape of these genomes. TEs can promote intra- and inter-specific variability in terms of genome structure, size, and transcriptional regulation, and their proliferation control can be tightly linked with DNA methylation or heterochromatin-associated histone modifications (12, 108–110).

Analyses of TE content in Zt469 and Za100 revealed that the two genomes vary in the overall proportion of TEs: 16.34% in Za100 compared to 17.91% in Zt469 (Fig. S8A). Comparing the composition of TE families in the two genomes, we found, in agreement with previous studies, a high proportion of LTR-retrotransposons compared to other TE families (Fig. S8B) (50). Regarding unitig 3 and unitig 9, although both accessory unitigs are enriched in TEs (Fig. 3 and 6), unitig 3 in Za100 shows a higher TE proportion (15.02% of TEs and approximately 0.38 Mb of the chromosome length) compared to unitig 9 in Zt469 (13.59% of TEs and approximately 0.23 Mb of the chromosome length) (Fig. S9A). In both unitigs 3 and 9, retrotransposons from the LINE order make up the largest portion of TEs (Fig. S9B), followed by other LTRs and DNA transposons, suggesting the enrichment of similar elements on the two homologous chromosomes in the two fungal species.

**Fig 7:**
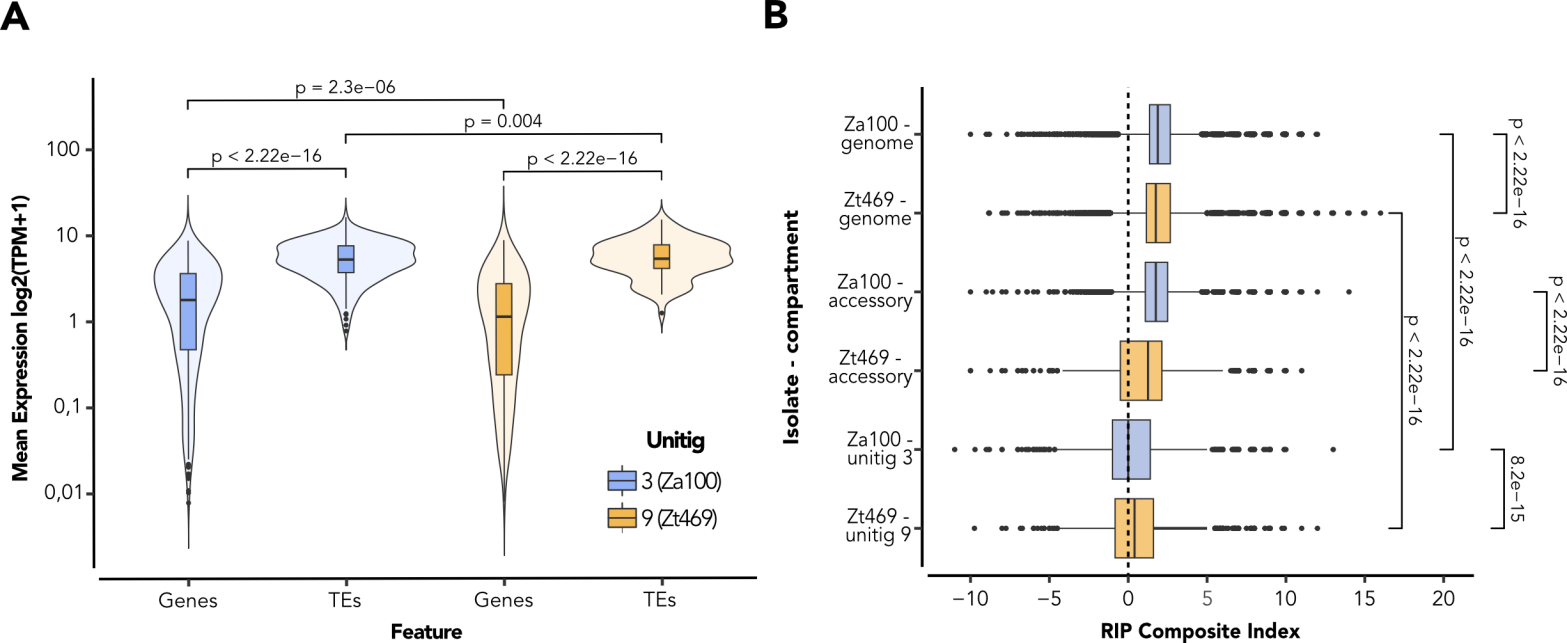
Unitig 3 and unitig 9 have high TE transcription activity and differential RIP mutation signatures. (**A**) Violin plots representing the mean expression levels in log2(TPM + 1) for both gene and TE features of unitig 3 in Za100 (violet color) and unitig 9 in Zt469 (orange color) during *in vitro* growth. *P*-values were calculated using pairwise Wilcoxon rank-sum tests. (**B**) Distribution of RIP composite indices calculated in 50 bp sliding windows per TE copy distributed genome-wide (top two boxes), per TE copy present on unitigs 3 and 9 (bottom two boxes), and per TE copy present in all the other accessory unitigs (middle boxes) for *Z. ardabiliae* Za100 (violet color) and *Z. tritici* Zt469 isolates (orange color). Vertical dashed line represents the threshold (0) above which composite index values indicate RIP signature. *P*-values were calculated using pairwise Wilcoxon rank-sum tests.

Interestingly, not only do the unitigs differ in TE proportion, but they also differ in the levels of expression of these genomic elements during *in vitro* growth. Although both unitigs show a high TE expression, a comparative analysis of TE expression based on RNA-seq data showed significantly higher levels of expression of TEs on unitig 3 in *Z. ardabiliae* compared to the expression of TEs on unitig 9 in *Z. tritici* (Wilcoxon rank-sum test, *P*-value = 0.004) (Fig. 7A). For both unitig 3 and unitig 9, expression levels of TEs were, in general, significantly higher than expression levels of genes within the same chromosome (Wilcoxon rank-sum test, *P*-value < 2.22e-16) (Fig. 7A). Based on these results, we hypothesized that unitig 3 and unitig 9 have a reduced efficacy of genome defense mechanisms regulating TE activity.

To further test this hypothesis, we assessed signatures of another genome defense mechanism known to be prominent in many ascomycetes, namely repeat-induced point mutations (111, 112). RIP signatures strongly correlate with a dinucleotide bias containing A and T at the sequence level (111). Thus, in order to quantify putative RIP-derived mutations, we scanned each genome individually and searched for dinucleotide bias mutations in duplicated sequences. Based on this, we calculated the RIP composite indices and determined the distribution of large RIP-affected regions in 1 kbp genomic windows using the RIPper software (95). At the genome-wide level, we observed that RIP signatures were present in 21.85% of the total genomic content of Za100 and 18.61% of the total genomic content in Zt469 (Table S9). LRARs, besides comprising extensive AT-rich regions, have an average size of 23,585 and 18,299 bp and affected a total of 8.2 and 6.9 Mbp in the genomes of Za100 and Zt469, respectively (Table S9). Intriguingly, the proportion of RIP mutations differed considerably between the two species at chromosome levels: the overall percentage of 1 kbp regions per unitig affected by RIP ranged from 2.72% to 85.52% in Za100 and from 7.22% to 31.5% in Zt469 (Fig. S10). RIP signatures specifically in unitig 3 and unitig 9 comprised 24.09% and 28.95% of the total unitig contents, respectively (Fig. S10). We also observed that unitig 3 and unitig 9 show a higher-than-expected composition of A and T nucleotides, corroborating the RIP signatures observed for these chromosomes (Fig. S11).

We also estimated RIP composite indices per 50 bp windows of each TE copy in the genomes of both Zt469 and Za100 and observed that, on average, TE copies were statistically more RIPped in unitig 9 of Zt469 compared to TEs in unitig 3 of Za100 (Wilcoxon rank-sum test, *P*-value = 8.2e-15; Fig. 7B). The same pattern was observed when including genome-wide TE copies (Fig. 7B). In contrast, TE copies located on other accessory unitigs in Zt469 (unitigs 18, 19, and 20) showed less RIP signatures than those located on the other accessory unitigs of Za100 (unitigs 25, 26, 28, 30, 31, and 34; Fig. 7B). Altogether, our results suggest that the efficacy of RIP has been higher for TEs on unitig 9 compared to unitig 3. The lower extent of H3K27me3 and the overall higher transcriptional activity of TEs in unitig 3 corroborate our deduction that unitig 3 of Za100 is enriched with active TEs.

### Gene expression in unitig 9 suggests a possible role during infection

We finally asked if unitig 9 in *Z. tritici* Zt469 has a functional relevance by analyzing gene and TE expression patterns during *in vitro* growth and during *in planta* infection. For expression profiling *in planta*, we analyzed stage-specific RNA-seq data sets based on four infection stages using previously generated transcriptome data (46). To determine the levels of expression in individual genes and TEs, we used the gene and TE models described previously and functionally annotated each gene using different tools (see Materials and Methods and Tables S2, S3, and S5). Expression levels, based on three biological replicates per condition, were compared using normalized read mappings to TPM in log2(TPM + 1). The percentage of reads that could be mapped to the Zt469 genome was, on average, 93.4% between replicates for *in vitro* growth and ranged from 2.3% to 76.5% between replicates and infection stages during *in planta* infection (Table S10).

At first, we focused on the overall expression levels of genes and TEs in unitig 9 and asked if the expression of these features was different between *in vitro* and *in planta* conditions. We observed a lower transcriptional activity of genes compared to TEs not only *in vitro* but also *in planta* (Fig. 3; Fig. S12 and Table S11). Previous transcriptome studies of *Z. tritici* during *in vitro* growth and *in planta* infection have shown that, on average, the expression levels of genes located on accessory chromosomes were up to 20-fold lower (in RPKM) than the expression levels of genes present on core chromosomes (32, 33). Our results corroborate these findings by demonstrating that genes located in unitig 9, and also in the other accessory unitigs 18, 19, and 20, were expressed on average from 1.6- to 2.1-fold lower levels, in log2(TPM + 1), than the genes located in the core chromosomes (unitigs 0–8 and 10–17; Tables S11 and S12).

**Fig 8:**
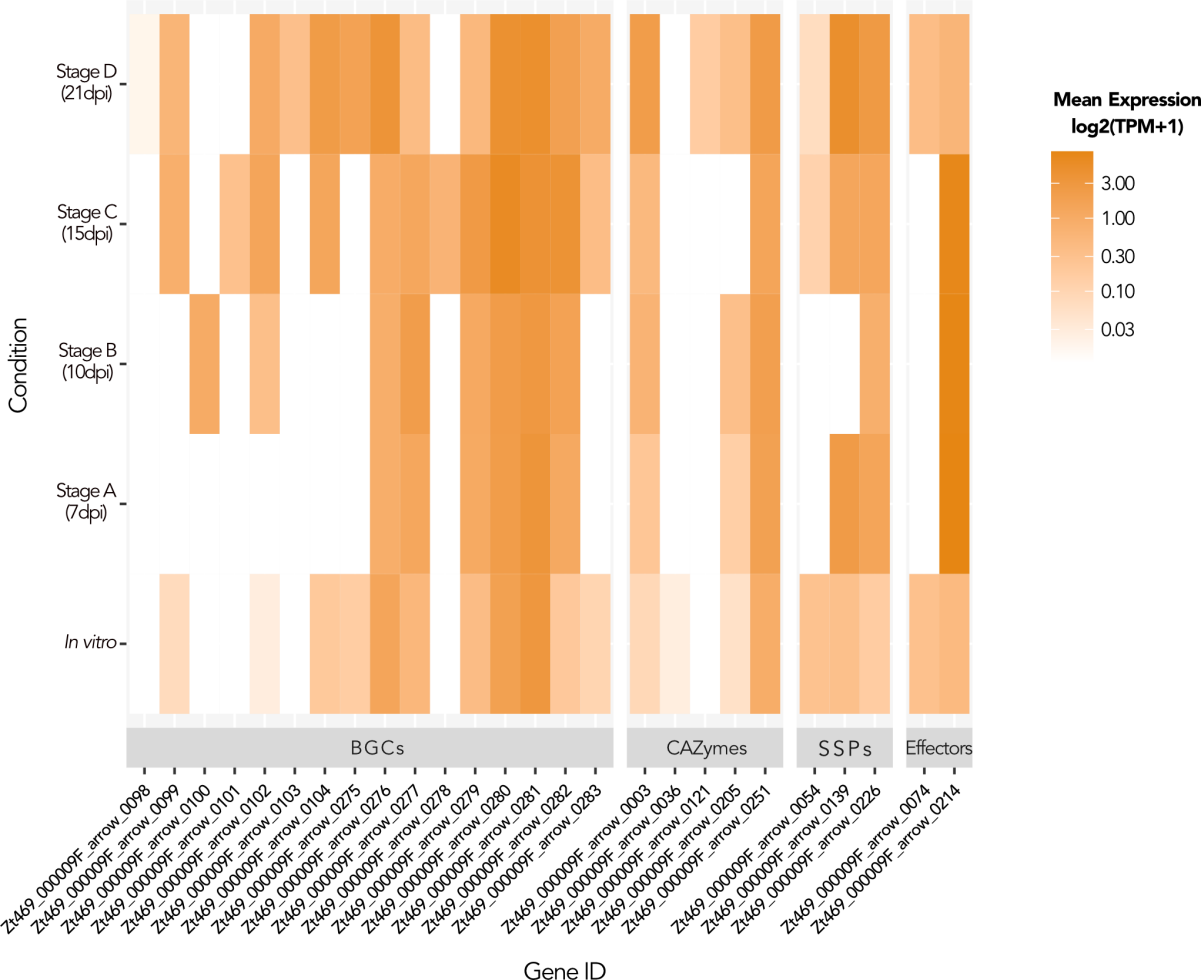
Gene categories involved in host-pathogen interactions show different levels of expression in unitig 9 during *in vitro* growth and *in planta* infection. Heatmaps showing mean expression levels in log2(TPM + 1) for each gene individually in unitig 9 belonging to four gene categories analyzed at different infection stages *in planta* and *in vitro*. White cells represent no transcription (TPM = 0). BGCs, biosynthetic gene clusters; CAZymes, carbohydrate-degrading enzymes; SSPs, small secreted proteins.

Next, we functionally annotated the gene models in the Zt469 genome and focused on the expression profile of specific gene categories in unitig 9 (see Materials and Methods and Tables S2, S3, and S5). We identified 26 genes with putative functions in host-pathogen interactions, including secreted proteins such as CAZymes (carbohydrate-degrading enzymes), effectors, and other SSPs, as well as secondary metabolites encoded by BGCs (113–116). Of these 26 genes in unitig 9 (Table S5), 16 genes showed no transcription (TPM = 0) in at least one condition (Fig. 8). Most of these 26 genes analyzed show no expression at infection stage “A” (7 dpi) followed by a low to intermediate expression, 0.03 < log2(TPM + 1) < 0.030, at infection stage “D” (21 dpi; Fig. 8). We also observed by differential expression analyses that the candidate effector gene Zt469_000009F_arrow_0214 was differentially expressed (*P*_adj_ < 0.01 and |log2 fold change ≥ 2|) between *in vitro* growth and the infection stages A–C, and between the infection stages C and D (Table S13). Taking these results together, we found that a small set of genes on unitig 9 shows different expression profiles during disease progression, suggesting a possible role in plant infection.

### Introgression tests suggest gene flow between *Aegilops*-infecting *Z. tritici* and *Z. ardabiliae*

Considering the synteny between unitig 9 of Zt469 and unitig 3 in Za100, we hypothesize that these accessory chromosomes may have been exchanged by introgression between *Aegilops*-infecting *Z. tritici* and *Z. ardabiliae* isolates. To test this hypothesis, we performed an ABBA-BABA test (102, 103), also known as Patterson’s *D*-score, among four taxa (P1, P2, P3, and O) to determine whether interspecific gene flow occurs. To this end, we included the *Aegilops*-infecting *Z. tritici* isolates Zt469 (with unitig 9) or Zt436 (without unitig 9) as P1; the wheat-infecting *Z. tritici* isolates Zt565 or Zt668 as P2; and the *Z. ardabiliae* isolates Za100 (with unitig 3) or Za98 (without unitig 3) as P3 (Fig. S1). We included *Aegilops*-infecting Z*. tritici* isolates with or without unitig 9 as well as *Z. ardabiliae* isolates with or without unitig 3 to evaluate whether gene flow only occurred between isolates harboring these newly described chromosomes or if signatures of introgression could be detected generally at the lineage and species levels. All tests were performed including the genome of the *Z. passerini* isolate Zpa796 as the outgroup (O).

Using the tree topologies, we observed significantly negative *D* statistic results (*Z*-score > |3|) for tests including *Aegilops*-infecting *Z. tritici* isolates with or without unitig 9, which indicates an excess of shared derived alleles between these Z*. tritici* isolates and *Z. ardabiliae* (Table S14). We also observed significantly negative *D* statistic results (*Z*-score > |3|) if *Z. ardabiliae* isolates with or without unitig 3 were included in the tests (Table S14). These results were consistent when we replaced Zt565 with another wheat-infecting *Z. tritici* isolate, Zt668 (Table S14). These findings suggest that interspecies hybridization occurs between *Aegilops*-infecting *Z. tritici* and *Z. ardabiliae* species, and it is not only restricted to isolates harboring the newly described chromosomes.

At last, we tried to access the relative age and possible introgression direction of the syntenic chromosomes in *Aegilops*-infecting *Z. tritici* and *Z. ardabiliae*. We calculated the levels of genomic variation in each chromosome using population data and used nucleotide diversity (π) per 1 kb windows as a proxy of age. Hereby, we hypothesized that an older chromosome would accumulate more mutations and therefore contain higher levels of nucleotide diversity compared to a younger chromosome. Using quality-filtered SNPs, we observed a statistically higher nucleotide diversity in unitig 9 of Zt469 than unitig 3 in Za100 (Wilcoxon rank-sum test, *P*-value < 0.001; Fig. S13). Based on our hypothesis, these results suggest that unitig 9 accumulated more polymorphisms and therefore may be older than unitig 3. This would imply that the chromosome originated first in *Z. tritici* and subsequently was transferred to *Z. ardabiliae* by introgression.

## DISCUSSION

Accessory chromosomes, frequently found in fungi, provide a source of genetic innovation. These chromosomes encode genes that are not essential to the organism and typically evolve with a reduced efficacy of purifying and background selection. Hereby, transposable elements can accumulate and contribute to the evolution of new genetic variants (1, 15, 17). The genetic mechanisms that can lead to the birth of a new accessory chromosome are poorly understood. In this study, we characterize a new and unique accessory chromosome in the fungal pathogen *Z. tritici*. Using a multi-omics approach, high-quality whole-genome assemblies, and gene and TE annotations from different isolates and species of *Zymoseptoria*, we uncover the most plausible scenario whereby the accessory chromosome has evolved. Our results confirm that recurrent interspecies hybridization occurs between pathogen species in natural vegetations and can serve as a route for accessory chromosome emergence.

Consistent with previous analyses on the genomic structure of *Zymoseptoria* species (20, 32, 33, 38, 39), the genome architecture in the analyzed *Zymoseptoria* isolates is also highly compartmentalized. We demonstrate that unitig 9 and the smaller unitigs 18, 19, and 20 in the *Aegilops*-infecting *Z. tritici* isolate Zt469 exhibit accessory chromosome hallmarks as described previously in the wheat-infecting *Z. tritici* and closely related *Zymoseptoria* species, including enrichment in heterochromatin methylation mark H3K27me3, PAV between isolates, and low levels of gene transcription *in vitro* and *in planta* (32, 33, 38). Similarly, in the *Z. ardabiliae* Za100 isolate, accessory unitigs, including the large unitig 3, showed characteristics that distinguish them from the core unitigs, e.g., PAV between isolates, low gene and high transposon densities, and low transcriptional gene activity. Considering the divergence observed between the *Aegilops*- and wheat-infecting *Z. tritici* isolates (46) and between the *Z. tritici* and *Z. ardabiliae* species (4, 26, 36, 37), these results corroborate previous findings that genome compartmentalization and accessory chromosomes are ancestral traits among *Zymoseptoria* species (38).

We observed interesting patterns regarding TE suppression on unitig 3 of Za100. One of the crucial roles of heterochromatin in eukaryotes is to prevent genome instability by silencing TE replication and spread (117). In several fungal plant pathogens, including *Zymoseptoria* species, enrichment of the facultative heterochromatin methylation mark H3K27me3 has been considered a hallmark of accessory chromosomes and can explain the overall transcriptional gene silencing observed for these chromosomes (16, 20, 118). H3K9me2/3, on the other hand, has been demonstrated to be enriched in repeat-rich regions with a direct impact on transposable element control and genome stability (107, 119–121). Transcriptional activation of TEs can result in their spread throughout the host genome, causing insertional mutations and chromosomal rearrangements (122–124). Moreover, several ascomycete fungi comprise a unique genome defense mechanism, known as RIP (repeat-induced point mutations), to mutate and thereby inactivate repetitive sequences (111, 112, 125). The genomes of both Zt469 and Za100 comprise intact copies of the DNA methyltransferase genes RID and DIM2, which are shown to be involved in *de novo* DNA methylation and RIP processes promoting C to T transitions (41, 126). This observation suggests that these fungi have active mechanisms for TE silencing.

Interestingly, although we observed that unitig 3 shows most of the expected features of an accessory chromosome, this unitig was not enriched with the heterochromatin methylation marks H3K27me3 and H3K9me3. We also observed that unitig 3 and the TEs that reside within this unitig have reduced RIP signatures when compared to most small and accessory unitigs in Za100 and to the syntenic unitig 9 in Zt469. In line with these results, we found that the levels of TE transcription in unitig 3 are high overall and even superior to the transcription of genes on the same unitig, as well as to the levels observed for unitig 9 in Zt469. Taking these results together, we conclude that unitig 3 lacks efficient silencing mechanisms, which may have resulted in the spread of TEs along the unitig and rapid sequence diversification. We note that the composition of dinucleotides suggests an excess of TA/AT dinucleotides on unitig 3 (Fig. S11), which may indicate an increasing rate of mutated TEs in this particular genome compartment. The relevance of distinct genome compartments in the maintenance of active TEs has also been demonstrated in flies. In male *Drosophila* flies, it was found that the “toxic” repeat-rich Y chromosomes act as reservoirs of actively transcribed TEs that may lead to reduced male fitness by deleterious TE mobilization and ineffective heterochromatin silencing of these repetitive elements (127, 128). Further analyses on Za100 TE activity and their genomic and epigenetic control *in vitro* and potentially *in planta* can shed light on the dynamics of TEs and their impact on *Z. ardabiliae* development and fitness.

Exchange of chromosomes between pathogen lineages or species through introgression can generate novel pathotypes and ultimately new pathogen species (129–131). Experimental evidence from *F. oxysporum* demonstrates that accessory (or lineage-specific) chromosomes can be horizontally transferred between distinct lineages by hyphal fusion and convert non-pathogenic strains into a pathogen in specific hosts (3, 132). Other reports have also shown that horizontal transfer of chromosomes between fungal strains can occur in *Colletotrichum gloeosporioides* (133), in *Alternaria alternata* (134), in the insect fungal pathogen *Metarhizium robertsii* (135), and in the cereal blast pathogen *Magnaporthe oryzae* (136)*.* In *Z. tritici*, it has been suggested that accessory chromosomes have originated via ancient horizontal transfer from an unknown donor followed by extensive recombination events (30). However, other studies have pointed to alternative mechanisms. One study demonstrated that a *Z. tritici* accessory chromosome emerged by non-disjunction duplication of core chromosomes followed by a degeneration process via breakage-fusion-bridge cycles and RIP on duplicated sequences (24). Here, we demonstrate that the introgression of chromosomes between *Zymoseptoria* species, followed by TE activation, may be an alternative mechanism whereby new accessory chromosomes can arise and diversify.

The relevance of introgression in accessory chromosome evolution is apparent from the comparison of genome sequences across diverse *Zymoseptoria* genomes. First of all, we found that the accessory unitig 9 in *Aegilops*-infecting *Z. tritici* is syntenic to another accessory chromosome (unitig 3) in the *Z. ardabiliae* species. The lack of further population data of *Z. ardabiliae* isolates harboring unitig 3 prevented us from exploring whether the chromosome itself represents an introgressed genomic segment, as well as determining the time and direction of introgression. However, we found evidence that genome-wide introgression signatures are present between *Aegilops*-infecting *Z. tritici* and *Z. ardabiliae* species through ABBA-BABA tests, which suggests that interspecies hybridization takes place and the transfer of chromosomes is likely to occur between these two species, particularly when considering their sympatry in grassland vegetations in the Middle East (26, 36, 37). Considering the findings we gathered so far, some hypothetical introgression scenarios can be made about the evolutionary origin of the novel accessory chromosomes in *Z. tritici* and *Z. ardabiliae* species.

In a first scenario, we consider that these newly described chromosomes were acquired independently from a common ancestral *Zymoseptoria* population and, since then, have been segregating only in *Aegilops*-infecting *Z. tritici* and *Z. ardabiliae* populations. This scenario could be supported by the host range and evolutionary history of the *Zymoseptoria* genus in the Middle East. It is possible that the newly described unitig 9 and unitig 3 persisted in wild grass-infecting populations of *Z. tritici* and *Z. ardabiliae* from the last common ancestral *Zymoseptoria* population that also infected wild grasses. This scenario is, however, incompatible with the unexpected sequence similarity of unitig 3 and unitig 9.

In a second scenario, chromosomal introgression could have happened from *Z. ardabiliae* into *Aegilops*-infecting *Z. tritici*. In this scenario, we may assume that unitig 3 is a chromosome that remained from ancestral populations and is being lost in the *Z. ardabiliae* species, resulting in the low frequency of the unitig among *Z. ardabiliae* isolates in our collection. The detrimental effects of active TEs (122–124) and the lower efficacy of genome defenses against TE replication (e.g., heterochromatin-associated methylation and RIP) on unitig 3 may have led to selection against this chromosome over *Z. ardabiliae* generations. The considerably lower proportion of TEs on the introgressed chromosome, however, argues against this scenario.

In a third introgression scenario, we consider the possibility that a recent chromosomal introgression happened from *Aegilops*-infecting *Z. tritici* isolates into the *Z. ardabiliae* species. This scenario can be supported by the low frequency of unitig 3 observed among our collection of *Z. ardabiliae* isolates spanning different years and locations, suggesting that this chromosome has not been established yet at the *Z. ardabiliae* species level. The higher degree of nucleotide diversity observed in unitig 9 compared to unitig 3 also suggests that unitig 9 in *Aegilops*-infecting *Z. tritici* isolates accumulated more polymorphisms and is therefore potentially older than unitig 3 in *Z. ardabiliae*. Moreover, we also observed high TE transcription activity, low enrichment of the heterochromatin-associated methylation marks (H3K27me3 and H3K9me3), and low RIP signatures in unitig 3 and in the TE copies that reside within this chromosome. This could further corroborate that unitig 3 has been recently introgressed and has since been acting as a reservoir of transcriptionally active TEs.

In light of our findings, we suggest that the third introgression scenario is the most parsimonious explanation for the origin and maintenance of the syntenic accessory chromosomes unitig 9 and unitig 3 in *Z. tritici* and *Z. ardabiliae* species. Further sampling of *Z. ardabiliae* isolates containing unitig 3 is required to better characterize the evolution of the newly acquired chromosome in this species.

### Conclusions

Accessory chromosomes have been demonstrated in a variety of eukaryotic species, including fungi, yet the evolutionary scenarios giving rise to these chromosomes are not well understood. Using the fungal plant pathogen *Z. tritici* as a model system, we provide evidence that accessory chromosomes can arise by interspecific hybridization and chromosome transfer. Increased activity of TEs on young accessory chromosomes can subsequently lead to chromosome length expansion and genetic divergence. Our findings provide valuable resources to further investigate the genetic mechanisms associated with the birth and establishment of a new accessory chromosome in a fungal genome. The occurrence of these chromosomes only in fungal species infecting wild grasses highlights the importance of wild pathosystems in the rapid genome evolution of fungal pathogens and illustrates a possible route whereby whole chromosomes may be introduced into related species infecting crops, like *Z. tritici* on wheat.

## Data Availability

All short-read genomic sequences analyzed during this study are publicly available in different repositories. For accession numbers, repositories, and references, please see Table S6. The Zt469 and Za100 assembled genomes can be found at https://doi.org/10.5281/zenodo.17592005. Gene and TE annotations for both Zt469 and Za100 are deposited at https://doi.org/10.5281/zenodo.17592005. *In planta* RNA-seq data sets for *Z. tritici* Zt469 from reference 46, as well as *in vitro* RNA-seq data sets and ChIP-seq data sets for both Za100 and Zt469, are available under the NCBI SRA BioProject accession number PRJNA1162778. The genome sequence of the reference *Z. tritici* isolate IPO323 (MYCGR v2.0) is available at NCBI under the RefSeq assembly GCF_000219625.1.

## SUPPLEMENTAL MATERIAL

Text S1 mbio.03468-25-s0001.docx Additional materials and methods.

Supplemental figures mbio.03468-25-s0002.pdf Figures S1 to S13.

Supplemental tables part 1 mbio.03468-25-s0003.xlsx Tables S1 to S7.

Supplemental tables part 2 mbio.03468-25-s0004.xlsx Tables S8 to S15.

ASM does not own the copyrights to Supplemental Material that may be linked to, or accessed through, an article. The authors have granted ASM a non-exclusive, world-wide license to publish the Supplemental Material files. Please contact the corresponding author directly for reuse.
