# Supplementary material for: Interspecies hybridization as a route of accessory chromosome origin in fungal pathogens
Source: mBio. 2026 Feb 24;17(4):e03468-25. doi: 10.1128/mbio.03468-25 (PMC13059725; doi:10.1128/mbio.03468-25)
Supplement: Text S1 — Additional materials and methods. [file mbio.03468-25-s0001.docx]

# Supplementary Text S1 for:

**Interspecies hybridization as a route of accessory chromosome origin in fungal pathogens**

Wagner C. Fagundes^1^*#, Mareike Möller^1,2^, Alice Feurtey^1,3,4^, Rune Hansen^1^, Janine Haueisen^1^, Fatemeh Salimi^5^, Alireza Alizadeh^6^, Eva H. Stukenbrock^1^#

^1^Environmental Genomics Group, Max Planck Institute for Evolutionary Biology, Plön & Christian-Albrechts University Kiel, Kiel, Germany

^2^Present Address: Research School of Biology, The Australian National University, Canberra, Australia

^3^ Present Address: Laboratory of Evolutionary Genetics, Institute of Biology, University of Neuchâtel, Neuchâtel, Switzerland

^4^ Present Address: Plant Pathology Group, Institute of Integrative Biology, ETH Zürich, Zürich, Switzerland

^5^Department of Plant Protection, College of Agriculture and Natural Resources, University of Tehran, Karaj, Iran

^6^Department of Plant Protection, Faculty of Agriculture, Azarbaijan Shahid Madani University, Tabriz, Iran

#Address correspondence to Eva H. Stukenbrock, [estukenbrock@bot.uni-kiel.de](mailto:estukenbrock@bot.uni-kiel.de) and Wagner C. Fagundes, [wagner.fagundes@tuebingen.mpg.de](mailto:wagner.fagundes@tuebingen.mpg.de)

*Present address: Adaptive Evolution of Filamentous Plant Pathogens, Max Planck Institute for Biology, Tübingen, Germany

**Additional material and methods**

**Genome assemblies filtering and telomeric repeats detection**

We filtered the *de novo* Zt469 and Za100 genome assemblies to exclude low quality contigs as performed previously (1, 2). To this end, we filtered out contigs with less than 1.5X and more than 2 times median read coverage as these may be unreliable due to missing data or might contain only repetitive DNA (1, 2) . The scripts used for the filtering of assemblies can be found at <https://gitlab.gwdg.de/alice.feurtey/genome_architecture_zymoseptoria>. For Zt469, these filtering steps removed 83 contigs from the raw genome assembly, resulting in a genome of 45 contigs, including a total of 40,151,964 bp (Supplementary Table S1). For Za100, filtering steps removed only one contig from the raw assembly, resulting in 38 contigs and an assembly size of 41,384,339 bp (Supplementary Table S1). Next, using the filtered genome assemblies, we detected the presence of canonical telomeric repeats (“CCCTAA”)(3) in the assembled contigs following scripts available from Feurtey et al. 2020 (2) and <https://gitlab.gwdg.de/alice.feurtey/genome_architecture_zymoseptoria>. Briefly, we first used Bowtie2 (4) to detect the canonical telomeric repeats in each assembly. We then converted the output of bowtie2 to BED format and used the software bedtools *sort* and *merge* v2.26.0 (5) to sort, merge and finally report the presence of more than six consecutive repeats at the contig ends (2, 3).

**Gene annotation pipeline**

Gene annotations for Zt469 and Za100 genomes followed a pipeline described in Feurtey et al. 2020 (2). Scripts used for these steps are available under https:// gitlab.gwdg.de/alice.feurtey/genome_architecture_zymoseptoria. Briefly, we first used GeneMark-ES for an *ab initio* prediction of gene models using the option “--fungus” (6) . Then, using RNA-seq data from fungal *in vitro* growth (described below), we increased the quality of *ab initio* gene predictions using the BRAKER1 pipeline (7). To this end, we mapped the quality-trimmed and masked RNA-seq reads to the newly assembled genomes using HISAT2 (8) and used the BRAKER1 pipeline to predict genes in each genome using the “--fungus” option (7). Quality-trimming and masking of low-quality nucleotides (Q<20) of RNA-seq reads are described under the “ChIP- and RNA-seq data analyses” methods. In a last step, we assembled the RNA-seq reads into gene transcripts using Trinity (9) and aligned these gene transcripts using PASA (10) . EVidence Modeler was then finally used to produce consensus gene models from the two independent predictions and the *de novo* assembled transcripts (11).

**Transposable elements identification and annotation**

We used different methods to obtain consensus sequences of TE families and annotate these elements in the Zt469 and Za100 genomes. First, we identified *de novo* consensus TE sequences in each genome using the *TEdenovo* tool from the REPET package (<https://urgi.versailles.inra.fr/Tools/REPET>) (12, 13) following the developer’s recommendations and default parameters as described in Feurtey et al. 2020 (2). Next, we used the tool MCHelper (14) and guidelines of Goubert et al. (2022) (15) to assist on the curation of *de novo* TE consensus sequences. In each *de novo* TE library, a maximum of 10 rounds of BLAST, Extract and Extend (BEE) processes, each with 1000 flanking bases at either end of the element, were performed in MCHelper with the options “-e 1000” and “-x 10” in full-automatic mode (“-a F -r A”) (14). To exclude possible false-positives, we filter out TEs that did not have at least 1 Full Length Fragment (FLF) with the option “-c 1” as well as those elements with homology to rRNAs or BUSCO genes from the Dothideomycete gene set with the option “-b dothideomycetes_odb10” in MCHelper. Consensus TE sequences with 60% or more simple sequence repeats (SSRs) were also excluded in the false-positive filtering step (14). We then manually excluded TE consensus sequences classified as “unconfirmed” or “incomplete” due to either lack or merging of coding domains leading to chimeric sequences. TE-Aid plots (15) implemented in MCHelper were used to visualize and manually inspect the “incomplete” TE sequences. As a second step, we merged our curated *de novo* TE consensus sequences with the pangenome *Z. tritici* TE library from Baril & Croll (2023) (16) using CD-Hit-Est v. 4.8.1 (17, 18). In this step, we clustered TEs to the family-level using the “80-80-80 rule” (i.e. ≥ 80% identity, ≥ 80% length, ≥ 80 bp) with the options “-d 0 -aS 0.8 -c 0.8 -G 0 -g 1 -b 500” (15, 16, 19) in CD-Hit-Est. After clustering, we manually checked each TE cluster and the representative of each cluster was selected to keep the sequence with the highest classification confidence based on TE-Aid (15) plots and previous TE classification described in Baril & Croll (2023) (16). At last, using the final, merged curated TE library, we annotated the Zt469 and Za100 genomes using RepeatMasker v. 4.1.8 (20) with the options “-gff” and “-cutoff 250” as well as “-nolow” and “-no_is” to ignore simple repeats and low-complexity regions. Annotated TEs shorter than 100bp were filtered out, and strand-adjacent TEs overlapping by more than 100 bp were merged using the tools *gff2bed* from BEDOPS (21) and bedtools *merge* v2.26.0 (5) with the options “-d -100 -s -c 6,10 -o distinct,distinct”. Each annotated TE element received an unique numeric identifier (e.g. “TE[n]”) for compatibility with downstream tools. The final TE annotations in BED format were used for all downstream analyses and can be found in the following Zenodo repository: <https://doi.org/10.5281/zenodo.13773246>.

**ChIP- and RNA-sequencing of *in vitro* cultures**

We used three biological replicates of the *Z. tritici* isolate Zt469 and *Z. ardabiliae* isolate Za100 grown *in vitro* for chromatin immunoprecipitation followed by sequencing (ChIP-seq) and RNA-sequencing. Cells from the same biological replicate were used for both RNA and ChIP DNA extractions. Fungal spores were grown on YMS plates for three to four days, harvested and resuspended in 6 mL of 1x PBS (137 mM NaCl, 2.7 mM KCl, 10 mM Na2HPO4, 1.8 mM KH2PO4). For RNA extraction, 1 mL of resuspended cells were centrifuged, ground in liquid nitrogen, and total RNA was extracted using TRIzol (Invitrogen, Karlsruhe, Germany) following the manufacturer’s instructions. Cleaned (DNAse-treated) RNA samples were sent to Admera Health (South Plainfield, NJ, USA) for library preparation. For ChIP, the remaining 5 mL of resuspended cells were crosslinked with 0.5% formaldehyde for 15 min at room temperature and quenched by adding 150 μL of 2.5 M glycine. Chromatin immunoprecipitation was performed as previously described (22, 23) using antibodies against H3K4me2 (#07–030, Merck Millipore), H3K9me3 (#39161, Active Motif) and H3K27me3 (#39155, Active Motif). ChIP-seq libraries were prepared with a modified version of the Next Ultra II DNA Library Prep Kit for Illumina (#E7645S, New England Biolabs). Sequencing of ChIP-seq and RNA-seq samples was performed on an Illumina HiSeq 3000, obtaining paired-end reads of 150 nt by Admera Health (South Plainfield, NJ, USA).

**ChIP-seq peak calling**

To assess the histone methylation landscape in the Zt469 and Za100 genomes during *in vitro* growth, we used the software HOMER (24) to detect peaks of methylation-enriched regions in our ChIP-seq mappings. Briefly, we first created “tag” directories for each replicate and histone modification using the command *makeTagDirectory* with the options “-unique” and “-fragLength given” to keep only uniquely aligned reads and to estimate fragment length of reads using the paired-end data. To call histone modification peaks, we used different settings for the euchromatic (H3K4me2) and heterochromatic (H3K9me3 and H3K27me3) histone methylation marks as performed previously (23). Briefly, for H3K4me2, we used the HOMER *findPeaks* command with the options “-style histone -region -size 1000 -minDist 500 -C 0”. For H3K9me3 and H3K27me3, the *findPeaks* command was used with the options “-style histone -region -size 2000 -minDist 2000 -C 0”. Peak callings were converted to BED format using the HOMER command *pos2bed.pl*.

**Gene expression analyses**

We analyzed the gene expression by mapping RNA-seq data generated from *in vitro* growth and *in planta* infection onto the Zt469 and Za100 assemblies. The Zt469 *in planta* RNA-seq data was obtained from a previous study (25) and represents data collected from Zt469-infected *Aegilops cylindrica* leaves at four infection stages ranging from early biotrophy to late necrotrophy [7, 10, 15 and 21 days-post inoculation (dpi)]. Quality-trimming and masking of RNA sequencing reads are described under the “ChIP- and RNA-seq data analyses” methods. We mapped the quality-trimmed and masked reads to each assembled genome using HISAT2 (8) with the options “--rna-strandness RF” and “--max-intronlen 1500”. We estimated the raw read counts per gene with htseq-count v2.0.2 (26) using the options “-f bam -m union --type=gene -r pos --idattr=Name --stranded=reverse”. Gene expression was then assessed as Transcript per Million (TPM) as described in Feurtey et al. 2020 (2). Briefly, we first normalized raw read counts with coding region length, resulting in the number of reads per kilobase (RPK). We then divided the RPK total counts per sample by 1 million to generate the “per million” scaling factor. At last, we divided the RPK values by the “per million” scaling factor, resulting in the TPM values (2). For each gene model, we obtained the TPM averages over the three sequenced replicates in each condition analyzed and reported values as log2(TPM+1). We considered genes to be expressed when log2(TPM+1) > 0 in at least one condition analyzed (*in vitro* or during infection stages *in planta*). Coding region lengths of each gene were obtained with the GenomicFeatures R package using the function “*exonsBy*” (27). For differential gene expression between infection stages and *in vitro* growth in *Z. tritici* Zt469, we used the R Bioconductor package DESeq2 v1.42.1 (28). We considered genes to be differentially expressed if the adjusted *p*-value (*p*_adj_) was ≤ 0.01 and |log2 fold change ≥ 2|.

**Transposable Element expression analyses**

We evaluated Transposable Elements (TEs) transcription activity in both Zt469 and Za100 genomes using the *in vitro* and *in planta* RNA-seq data. Raw RNA-seq reads were trimmed, quality filtered and masked as described in the “ChIP- and RNA-seq data analyses” methods. Quality-trimmed and masked reads were mapped to each respective genome assembly using HISAT2 (8) with the option “--no-mixed”. Then, we accessed the TE transcription *in vitro* and *in planta* for each replicate and each infection stage individually. To this end, read count tables were generated using the *TEcount* function of TEtranscript pipeline with the options “--mode multi” and “--stranded reverse” (29, 30), and levels of expression were reported using the Transcript per Million (TPM) normalization as described above. The length of each TE was calculated with the GenomicFeatures R package using the function “*exonsBy*”, in which the entire element was treated as a unique exon (27).

**Pulsed-Field Gel Electrophoresis (PFGE) and Southern blot analyses**

For PFGE analyses, we used chromosomal DNA of *Hansenula wingei* (Bio-Rad, Munich, Germany) as standard size marker for mid-size chromosomes (1 to 3.1 Mb). PFGE run settings were followed as described previously (31). Gels were stained with ethidium bromide solution (1 mg/ml ethidium bromide in H2O) for 30 min and chromosomal bands were detected with the GelDocTM XR+ system (Bio-Rad). We performed Southern blot analysis as described previously (22) using a DIG (digoxigenin)-labeled probe generated by the PCR digoxigenin labeling Mix (Roche, Mannheim, Germany) following the manufacturer’s instructions. Probe primers were designed with the Primer-BLAST (32) and Geneious v.2020.1.2 software (https://www.geneious.com/home/). A list of primers can be found in the Supplementary Table S15.

**Introgression analyses**

In order to compute the ABBA-BABA tests, whole-genome paired-end sequencing reads (2x150bp) were first trimmed using Trimmomatic v 0.39 (33) with the following parameters: LEADING:20 TRAILING:20 SLIDINGWINDOW:5:20 MINLEN:50. Trimmed reads were then aligned to the *Z. tritici* IPO323 reference genome (34) using the short-read aligner bwa-mem v. 0.7.17 (35) . For these tests, we only aligned reads to the core genome (chromosomes 1 to 13) of *Z. tritici* to avoid biases due to presence-absence variation of accessory chromosomes. Alignments were converted to binary files (.bam) and sorted using SAMtools v. 1. 13 (36). Read duplicates and read groups were determined using Picard tools v. 2.26.2 (<https://broadinstitute.github.io/picard/>) and alignment files were then finally used as input in the “D-stat” module from the ANGSD package (Analysis of Next Generation Sequencing Data; <https://www.popgen.dk/angsd/index.php/Abbababa>) (37). We used a block size of 15kb (“-blockSize 15000”) to account for possible linkage disequilibrium (LD) blocks based on previous reports of LD decay in *Z. tritici* populations (25, 38, 39) and only kept sites for which we had data from all individuals (“-minInd 4”) with a minimum mapping quality score of 20 (“-minMapQ 20”).

**Variant calling**

In order to analyze nucleotide diversity per chromosome, we generated Variant Calling Format (VCF) files using short-read population data of *Aegilops*-infecting *Z. tritici* and *Z. ardabiliae* isolates obtained from previous studies (25, 40–42). Only the 11 *Aegilops*-infecting *Z. tritici* and 3 *Z. ardabiliae* isolates with reads mapped to unitig 9 (in Zt469) and unitig 3 (in Za100), respectively, were used for these analyses. First, we trimmed whole-genome paired-end sequencing reads (2x150bp) using Trimmomatic v 0.39 (33) with the following parameters: LEADING:20 TRAILING:20 SLIDINGWINDOW:5:20 MINLEN:50. Trimmed reads were then aligned to the *Aegilops*-infecting *Z. tritici* Zt469 (for *Aegilops*-infecting *Z. tritici* isolates) and *Z. ardabiliae* Za100 (for *Z. ardabiliae* isolates) reference genomes using the short-read aligner bwa-mem v. 0.7.17 (35) with default settings. Conversion, sorting, merging, and indexing of alignment files were performed using SAMtools v. 1. 13 (36). PCR duplicates and read groups were determined using Picard tools v. 2.26.2 (https://broadinstitute.github.io/picard/). Single Nucleotide Polymorphism (SNP) calling, genotyping and variant filtration were performed using the Genome Analysis Toolkit (GATK) v. 4.1.4.1 (43) for each species individually. SNPs were hard-filtered using the GATK *VariantFiltration* and *SelectVariants* tools. We first filtered low-depth genotypes using the following parameters: --genotype-filter-expression "DP < 3", --set-filtered-genotype-to-no-call and --genotype-filter-name "low_depth". Then, the following filter conditions were applied: DP > 1000.0 (for *Aegilops*-infecting *Z. tritici*) and DP > 2000.0 (for *Z.ardabiliae*) ; QD < 20.0; MQ < 50.0; FS > 20.0; ReadPosRankSum, MQRankSum, and BaseQRankSum between -2 and 2. At last, we kept only biallelic SNPs with a genotyping rate of ≥ 90% for each species using VCFtools v 0.1.13 (44) with the options “--min-alleles 2 --max-alleles 2 --max-missing 0.9”.

**Homology-based detection of unitig 9 in a worldwide collection of *Z. tritici* isolates**

We accessed the presence of unitig 9 in a worldwide panel of *Z. tritici* isolates consisting of more than 1000 isolates spanning different geographical regions and collection years published by Feurtey et al. 2023 (45). To this end, we performed nucleotide BLAST searches (blastn) (46) using the chromosome sequence of unitig 9 from Zt469 as a database and each of the 1291 draft *Z. tritici* genome assemblies available from (<https://doi.org/10.5281/zenodo.13645366>; (45) as query sequences. As comparison and controls, we also used the long-read Za100 genome assembly and as well as a *de novo* draft genome of Za100 assembled using short-read Illumina data published previously (42). We assembled the draft Za100 genome assembly using SPAdes v. 3.14.1 (47) with the same settings as (45) (“--careful" option). At last, we concatenated all BLAST hit tables and plot the number of isolates per hit length bin (in base pairs) using R (48).

**References**

1. Plissonneau C, Stürchler A, Croll D. 2016. The evolution of orphan regions in genomes of a fungal pathogen of wheat. mBio 7.

2. Feurtey A, Lorrain C, Croll D, Eschenbrenner C, Freitag M, Habig M, Haueisen J, Möller M, Schotanus K, Stukenbrock EH. 2020. Genome compartmentalization predates species divergence in the plant pathogen genus Zymoseptoria. BMC Genomics 21:588.

3. Fulnečková J, Ševčíková T, Fajkus J, Lukešová A, Lukeš M, Vlček Č, Lang BF, Kim E, Eliáš M, Sýkorová E. 2013. A Broad Phylogenetic Survey Unveils the Diversity and Evolution of Telomeres in Eukaryotes. Genome Biol Evol 5:468–483.

4. Langmead B, Salzberg SL. 2012. Fast gapped-read alignment with Bowtie 2. Nature Methods 9:357–359.

5. Quinlan AR, Hall IM. 2010. BEDTools: A flexible suite of utilities for comparing genomic features. Bioinformatics 26:841–842.

6. Ter-Hovhannisyan V, Lomsadze A, Chernoff YO, Borodovsky M. 2008. Gene prediction in novel fungal genomes using an ab initio algorithm with unsupervised training. Genome Research 18:1979–1990.

7. Hoff KJ, Lange S, Lomsadze A, Borodovsky M, Stanke M. 2016. BRAKER1: Unsupervised RNA-Seq-based genome annotation with GeneMark-ET and AUGUSTUS. Bioinformatics 32:767–769.

8. Kim D, Langmead B, Salzberg SL. 2015. HISAT: A fast spliced aligner with low memory requirements. Nature Methods 12:357–360.

9. Grabherr MG, Haas BJ, Yassour M, Levin JZ, Thompson DA, Amit I, Adiconis X, Fan L, Raychowdhury R, Zeng Q, Chen Z, Mauceli E, Hacohen N, Gnirke A, Rhind N, Di Palma F, Birren BW, Nusbaum C, Lindblad-Toh K, Friedman N, Regev A. 2011. Full-length transcriptome assembly from RNA-Seq data without a reference genome. Nature Biotechnology 29:644–652.

10. Haas BJ, Delcher AL, Mount SM, Wortman JR, Smith RK, Hannick LI, Maiti R, Ronning CM, Rusch DB, Town CD, Salzberg SL, White O. 2003. Improving the Arabidopsis genome annotation using maximal transcript alignment assemblies. Nucleic Acids Research 31:5654–5666.

11. Haas BJ, Salzberg SL, Zhu W, Pertea M, Allen JE, Orvis J, White O, Robin CR, Wortman JR. 2008. Automated eukaryotic gene structure annotation using EVidenceModeler and the Program to Assemble Spliced Alignments. Genome Biology 9:1–22.

12. Quesneville H, Bergman CM, Andrieu O, Autard D, Nouaud D, Ashburner M, Anxolabehere D. 2005. Combined evidence annotation of transposable elements in genome sequences. PLoS Computational Biology 1:0166–0175.

13. Flutre T, Duprat E, Feuillet C, Quesneville H. 2011. Considering transposable element diversification in de novo annotation approaches. PLoS ONE 6.

14. Orozco-Arias S, Sierra P, Durbin R, González J. 2024. MCHelper automatically curates transposable element libraries across eukaryotic species. Genome Res 34:2256–2268.

15. Goubert C, Craig RJ, Bilat AF, Peona V, Vogan AA, Protasio AV. 2022. A beginner’s guide to manual curation of transposable elements. Mobile DNA 13:1–19.

16. Baril T, Croll D. 2023. A pangenome-guided manually curated library of transposable elements for Zymoseptoria tritici. BMC Research Notes 16:335.

17. Fu L, Niu B, Zhu Z, Wu S, Li W. 2012. CD-HIT: accelerated for clustering the next-generation sequencing data. Bioinformatics 28:3150–3152.

18. Li W, Godzik A. 2006. Cd-hit: a fast program for clustering and comparing large sets of protein or nucleotide sequences. Bioinformatics 22:1658–1659.

19. Wicker T, Sabot F, Hua-Van A, Bennetzen JL, Capy P, Chalhoub B, Flavell A, Leroy P, Morgante M, Panaud O, Paux E, SanMiguel P, Schulman AH. 2007. A unified classification system for eukaryotic transposable elements. Nature Reviews Genetics 8:973–982.

20. Smit, AFA; Hubley, R; Green, P. 2013. RepeatMasker Open-4.0.

21. Neph S, Kuehn MS, Reynolds AP, Haugen E, Thurman RE, Johnson AK, Rynes E, Maurano MT, Vierstra J, Thomas S, Sandstrom R, Humbert R, Stamatoyannopoulos JA. 2012. BEDOPS: high-performance genomic feature operations. Bioinformatics 28:1919–1920.

22. Soyer JL, Möller M, Schotanus K, Connolly LR, Galazka JM, Freitag M, Stukenbrock EH. 2015. Chromatin analyses of Zymoseptoria tritici : Methods for chromatin immunoprecipitation followed by high-throughput sequencing (ChIP-seq). Fungal Genetics and Biology 79:63–70.

23. Möller M, Schotanus K, Soyer JL, Haueisen J, Happ K, Stralucke M, Happel P, Smith KM, Connolly LR, Freitag M, Stukenbrock EH. 2019. Destabilization of chromosome structure by histone H3 lysine 27 methylation. PLOS Genetics 15:e1008093.

24. Heinz S, Benner C, Spann N, Bertolino E, Lin YC, Laslo P, Cheng JX, Murre C, Singh H, Glass CK. 2010. Simple Combinations of Lineage-Determining Transcription Factors Prime cis-Regulatory Elements Required for Macrophage and B Cell Identities. Molecular Cell 38:576–589.

25. Fagundes WC, Hansen R, Barrera ICR, Caliebe F, Feurtey A, Haueisen J, Salimi F, Alizadeh A, Stukenbrock EH. 2024. Host specialization defines the emergence of new fungal plant pathogen populations. bioRxiv https://doi.org/10.1101/2024.09.30.615799.

26. Anders S, Pyl PT, Huber W. 2015. HTSeq-A Python framework to work with high-throughput sequencing data. Bioinformatics 31:166–169.

27. Lawrence M, Huber W, Pagès H, Aboyoun P, Carlson M, Gentleman R, Morgan MT, Carey VJ. 2013. Software for Computing and Annotating Genomic Ranges. PLoS Computational Biology 9:1–10.

28. Love MI, Huber W, Anders S. 2014. Moderated estimation of fold change and dispersion for RNA-seq data with DESeq2. Genome Biology 15:1–21.

29. Jin Y, Tam OH, Paniagua E, Hammell M. 2015. TEtranscripts: A package for including transposable elements in differential expression analysis of RNA-seq datasets. Bioinformatics 31:3593–3599.

30. Jin Y, Hammell M. 2018. Analysis of RNA-Seq Data Using TEtranscripts, p. 153–167. *In* Wang, Y, Sun, M (eds.), Transcriptome Data Analysis: Methods and Protocols. Springer New York, New York, NY.

31. Möller M, Habig M, Freitag M, Stukenbrock EH. 2018. Extraordinary Genome Instability and Widespread Chromosome Rearrangements During Vegetative Growth. Genetics 210:517–529.

32. Ye J, Coulouris G, Zaretskaya I, Cutcutache I, Rozen S, Madden TL. 2012. Primer-BLAST: a tool to design target-specific primers for polymerase chain reaction. BMC bioinformatics 13:134.

33. Bolger AM, Lohse M, Usadel B. 2014. Trimmomatic: A flexible trimmer for Illumina sequence data. Bioinformatics 30:2114–2120.

34. Goodwin SB, M’Barek SB, Dhillon B, Wittenberg AHJ, Crane CF, Hane JK, Foster AJ, van der Lee TAJ, Grimwood J, Aerts A, Antoniw J, Bailey A, Bluhm B, Bowler J, Bristow J, van der Burgt A, Canto-Canché B, Churchill ACL, Conde-Ferràez L, Cools HJ, Coutinho PM, Csukai M, Dehal P, de Wit P, Donzelli B, van de Geest HC, van Ham RCHJ, Hammond-Kosack KE, Henrissat B, Kilian A, Kobayashi AK, Koopmann E, Kourmpetis Y, Kuzniar A, Lindquist E, Lombard V, Maliepaard C, Martins N, Mehrabi R, Nap JPH, Ponomarenko A, Rudd JJ, Salamov A, Schmutz J, Schouten HJ, Shapiro H, Stergiopoulos I, Torriani SFF, Tu H, de Vries RP, Waalwijk C, Ware SB, Wiebenga A, Zwiers LH, Oliver RP, Grigoriev IV, Kema GHJ. 2011. Finished genome of the fungal wheat pathogen Mycosphaerella graminicola reveals dispensome structure, chromosome plasticity, and stealth pathogenesis. PLoS Genetics 7.

35. Li H, Durbin R. 2009. Fast and accurate short read alignment with Burrows-Wheeler transform. Bioinformatics 25:1754–1760.

36. Danecek P, Bonfield JK, Liddle J, Marshall J, Ohan V, Pollard MO, Whitwham A, Keane T, McCarthy SA, Davies RM, Li H. 2021. Twelve years of SAMtools and BCFtools. GigaScience 10:1–4.

37. Sand Korneliussen T, Albrechtsen A, Nielsen R. 2014. ANGSD: Analysis of Next Generation Sequencing Data.

38. Croll D, Lendenmann MH, Stewart E, McDonald BA. 2015. The impact of recombination hotspots on genome evolution of a fungal plant pathogen. Genetics 201:1213–1228.

39. Hartmann FE, Sánchez-Vallet A, McDonald BA, Croll D. 2017. A fungal wheat pathogen evolved host specialization by extensive chromosomal rearrangements. ISME Journal 11:1189–1204.

40. Stukenbrock EH, Bataillon T, Dutheil JY, Hansen TT, Li R, Zala M, McDonald BA, Wang J, Schierup MH. 2011. The making of a new pathogen: Insights from comparative population genomics of the domesticated wheat pathogen Mycosphaerella graminicola and its wild sister species. Genome Research 21:2157–2166.

41. Stukenbrock EH, Christiansen FB, Hansen TT, Dutheil JY, Schierup MH. 2012. Fusion of two divergent fungal individuals led to the recent emergence of a unique widespread pathogen species. Proceedings of the National Academy of Sciences 109:10954–10959.

42. Stukenbrock EH, Dutheil JY. 2018. Fine-Scale Recombination Maps of Fungal Plant Pathogens Reveal Dynamic Recombination Landscapes and Intragenic Hotspots. Genetics 208:1209–1229.

43. McKenna A, Hanna M, Banks E, Sivachenko A, Cibulskis K, Kernytsky A, Garimella K, Altshuler D, Gabriel S, Daly M, DePristo MA. 2010. The genome analysis toolkit: A MapReduce framework for analyzing next-generation DNA sequencing data. Genome Research 20:1297–1303.

44. Danecek P, Auton A, Abecasis G, Albers CA, Banks E, DePristo MA, Handsaker RE, Lunter G, Marth GT, Sherry ST, McVean G, Durbin R. 2011. The variant call format and VCFtools. Bioinformatics 27:2156–2158.

45. Feurtey A, Lorrain C, McDonald MC, Milgate A, Solomon PS, Warren R, Puccetti G, Scalliet G, Torriani SFF, Gout L, Marcel TC, Suffert F, Alassimone J, Lipzen A, Yoshinaga Y, Daum C, Barry K, Grigoriev IV, Goodwin SB, Genissel A, Seidl MF, Stukenbrock EH, Lebrun M-H, Kema GHJ, McDonald BA, Croll D. 2023. A thousand-genome panel retraces the global spread and adaptation of a major fungal crop pathogen. Nature Communications 14:1059.

46. Camacho C, Coulouris G, Avagyan V, Ma N, Papadopoulos J, Bealer K, Madden TL. 2009. BLAST+: architecture and applications. BMC bioinformatics 10:421.

47. Bankevich A, Nurk S, Antipov D, Gurevich AA, Dvorkin M, Kulikov AS, Lesin VM, Nikolenko SI, Pham S, Prjibelski AD, Pyshkin AV, Sirotkin AV, Vyahhi N, Tesler G, Alekseyev M a., Pevzner P a. 2012. SPAdes: A New Genome Assembly Algorithm and Its Applications to Single-Cell Sequencing. Journal of Computational Biology 19:455–477.

48. Team RC. 2013. R: A language and environment for statistical computing.
