# Supplementary material for: Interspecies hybridization as a route of accessory chromosome origin in fungal pathogens
Source: mBio. 2026 Feb 24;17(4):e03468-25. doi: 10.1128/mbio.03468-25 (PMC13059725; doi:10.1128/mbio.03468-25)
Supplement: Supplemental figures — Figures S1 to S13. [file mbio.03468-25-s0002.pdf]

Supplementary Figures

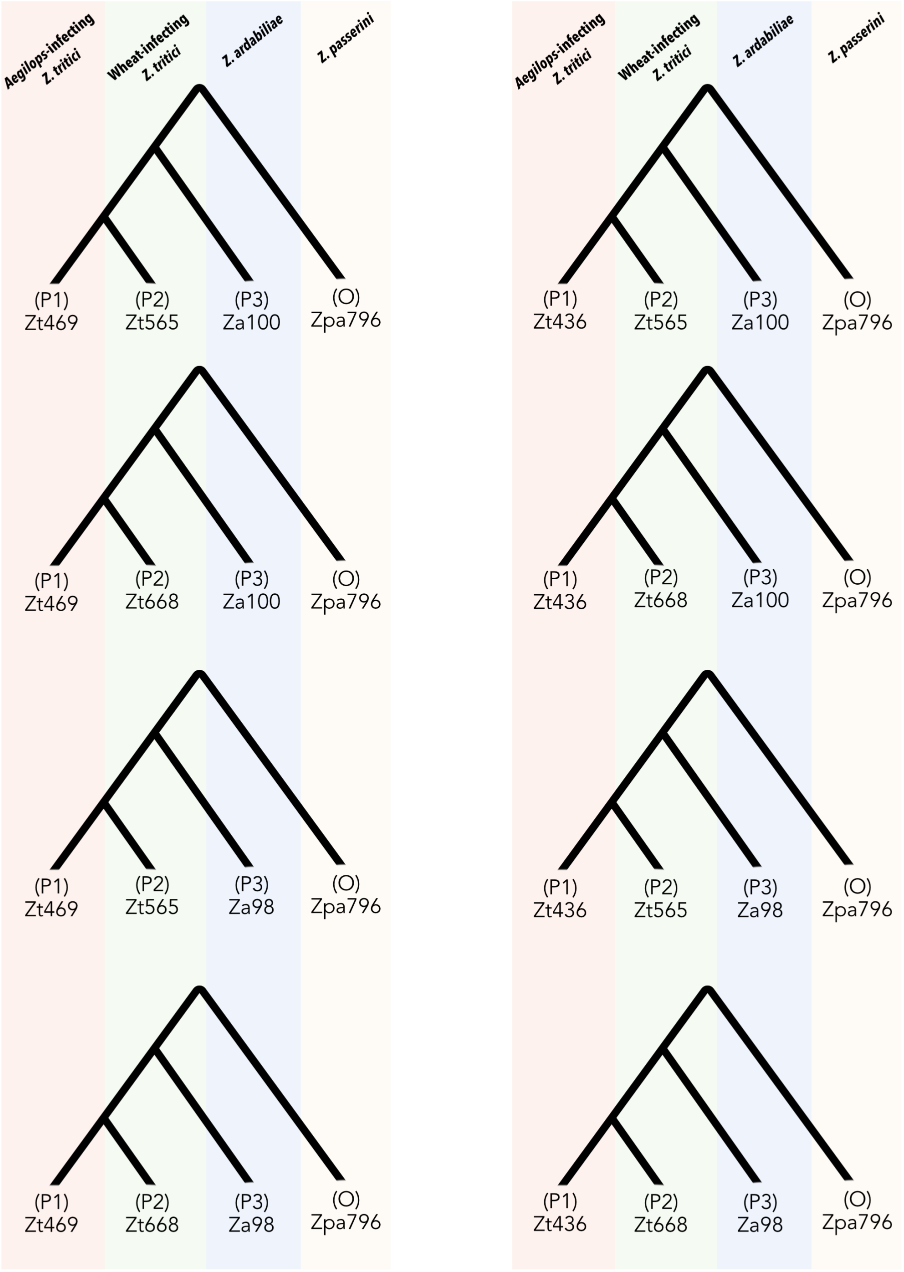

Figure S1. Tree topologies used in ABBA-BABA tests. ABBA-BABA tests were performed among four taxa (P1, P2, P3, O) in eight tree topologies. We included the *Aegilops*-infecting *Z. tritici* isolates Zt469 (with unitig 9) or Zt436 (without unitig 9) as P1; the wheat-infecting *Z. tritici* isolates Zt565 or Zt668 as P2; and the *Z. ardabiliae* isolates Za100 (with unitig 3) or Za98 (without unitig 3) as P3. All tests included the genome of the *Z. passerini* isolate Zpa796 as outgroup (O).

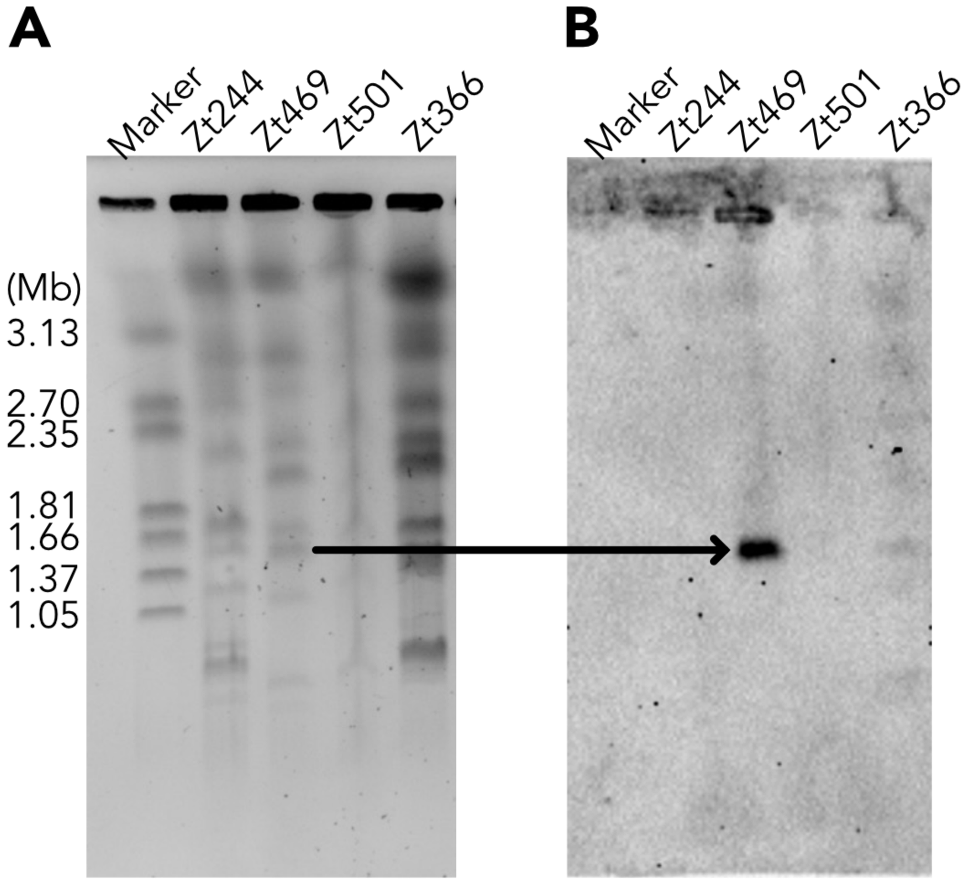

Figure S2. Presence of unitig 9 in Zt469 is confirmed by PFGE and Southern blot analyses. To validate the presence of unitig 9 at the expected assembly size in Zt469, we performed Pulsed- Field Gel Electrophoresis (PFGE) **(A)** followed by Southern blot analyses **(B)** in non-protoplast plugs of this isolate and in the reference *Z. tritici* isolate IPO323 (lab strain ID Zt244); in the wheat-infecting *Z. tritici* isolate Zt10 (strain Zt366); and in the *Aegilops*-infecting *Z. tritici* isolate Zt501. A southern blot probe of 1.5 kb was designed to hybridize specifically in unitig 9 of Zt469 (Supplementary Table S15). Probe hybridization was detected at the expected unitig 9 size (~1.6Mb; **B**). Chromosomal DNA of *Hansenula wingei* (Bio-Rad, Munich, Germany) was used as a standard size marker for PFGE.

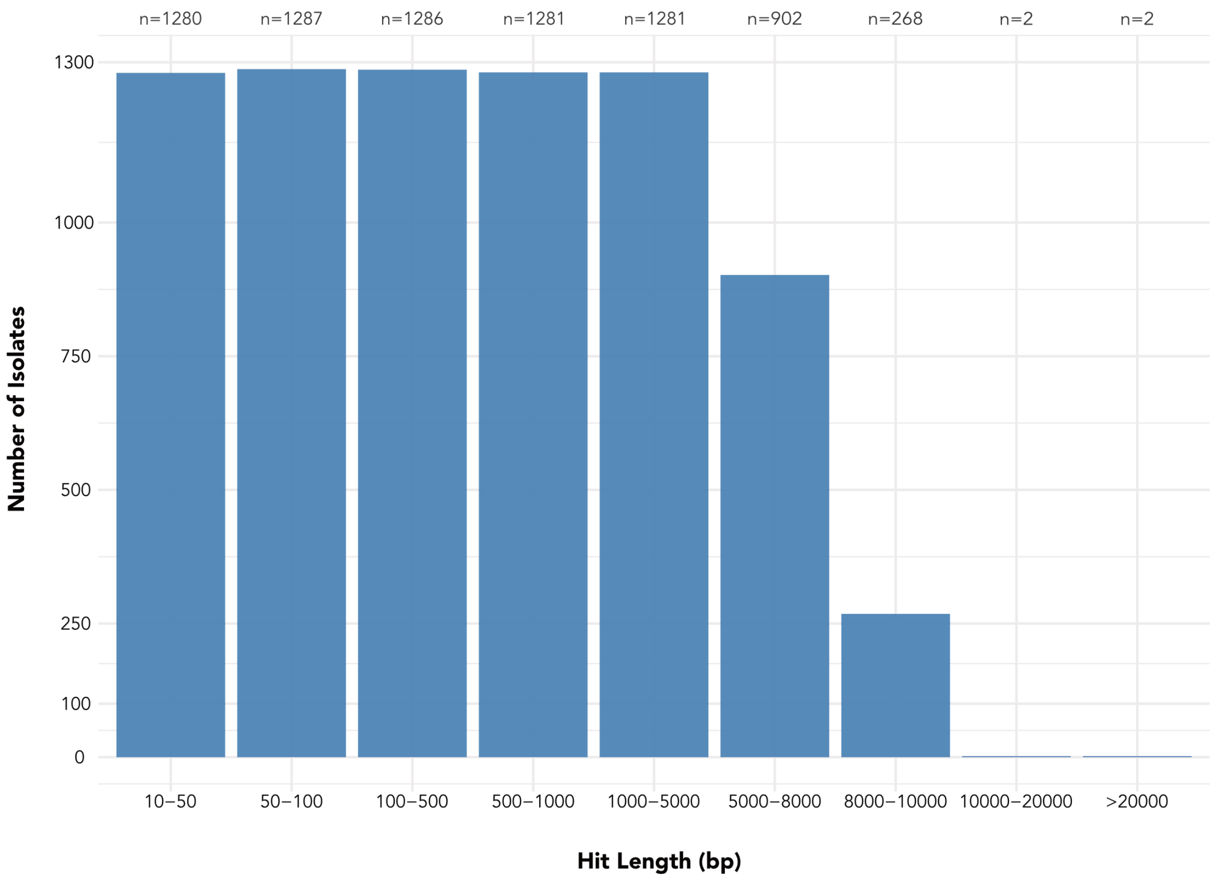

Figure S3. Homology-based detection of unitig 9 in a thousand-genome panel confirms its absence in wheat-infecting *Z. tritici* isolates. Summary of nucleotide BLAST (blastn) hits per hit length bin using each of the 1291 wheat-infecting *Z. tritici* genome assemblies from (Feurtey, A., Lorrain, C., McDonald, M.C. et al. A thousand-genome panel retraces the global spread and adaptation of a major fungal crop pathogen. Nat Commun 14, 1059 (2023). https://doi.org/10.1038/s41467-023-36674-y) as queries and unitig 9 chromosome sequence as a database. Only the newly assembled genomes from the *Z. ardabiliae* Za100 isolate (long-read and draft short-read assemblies, n=2; see Supplementary Text S1 and Supplementary Table S6) had hits larger than 10kb in length.

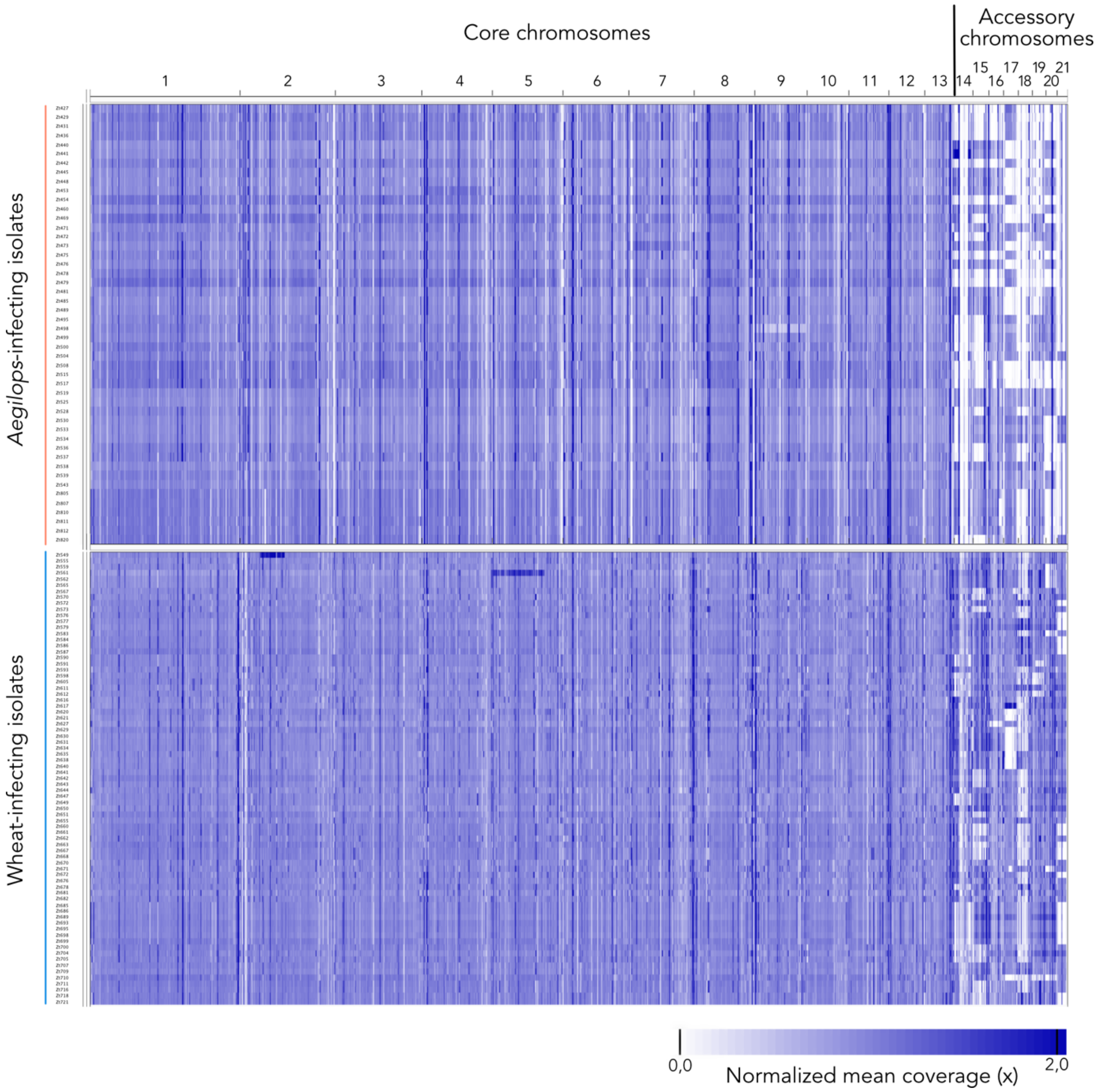

Figure S4. ***Aegilops*****-infecting** ***Z. tritici*** **isolates show high PAV of accessory chromosomes**. Chromosome presence-absence variation (PAV) in host-diverging *Z. tritici* populations was analyzed by read mapping to the *Z. tritici* IPO323 reference genome. Heatmap represents normalized mean coverage of reads mapped to each position of the reference genome in bins of 10 bp. Darker colors represent regions of higher coverage as e.g. repetitive elements or duplications.

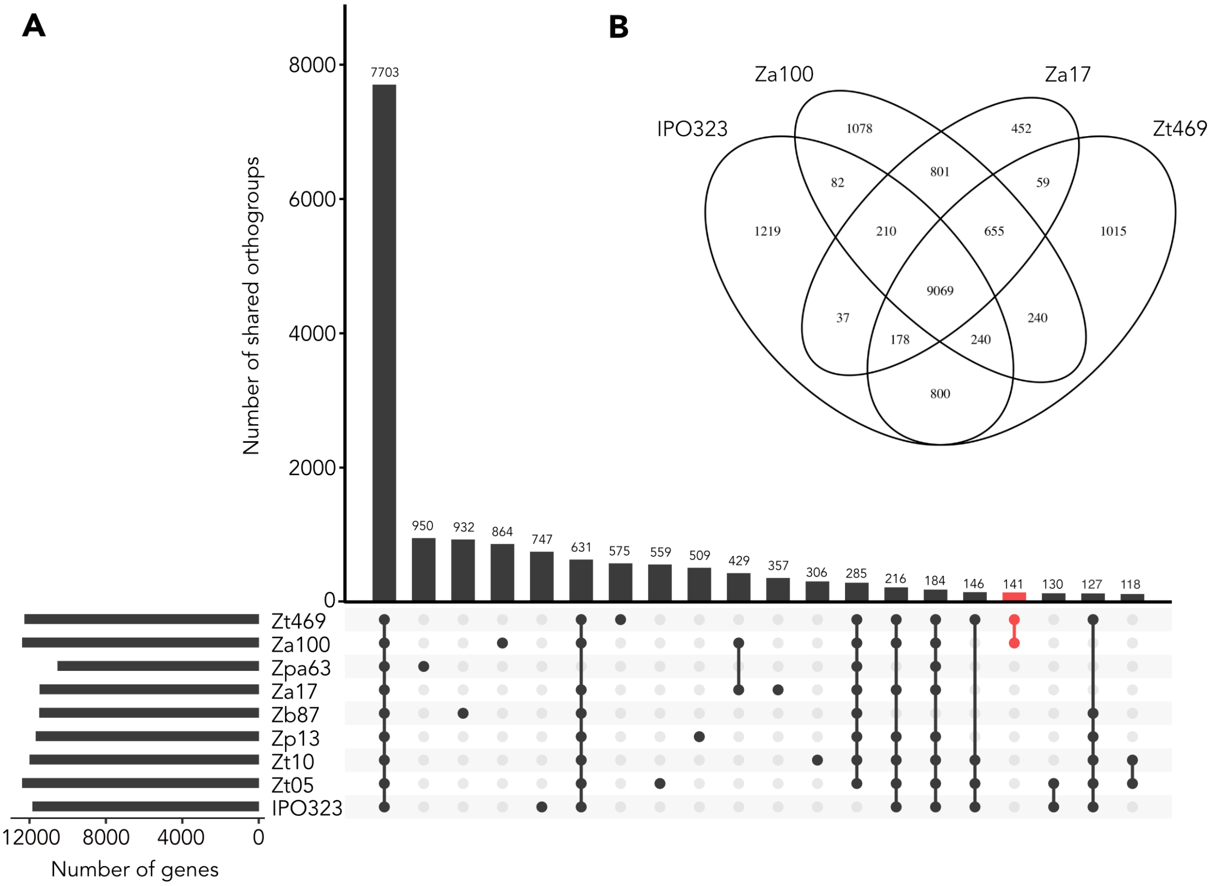

Figure S5. A large number of orthologs is shared between Zt469 and Za100 isolates. (A) Upset plot showing the number of orthogroups shared between the nine *Zymoseptoria* species genomes analyzed. Only intersects with more than 100 orthogroups are displayed. Number of orthogroups shared between Zt469 and Za100 are highlighted in red. **(B)** Venn diagram summarizing the orthogroups exclusively shared between the *Z. tritici* isolates IPO323 and Zt469 and *Z. ardabiliae* isolates Za17 and Za100.

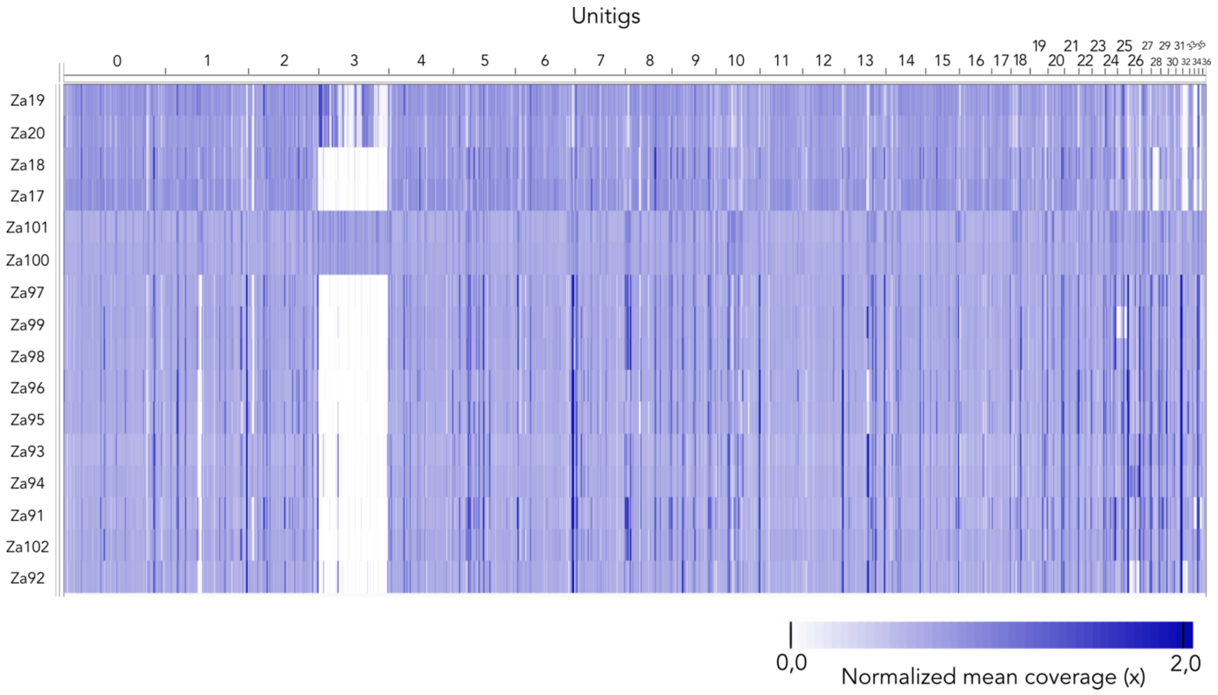

Figure S6. Unitig 3 shows presence-absence variation in *Z. ardabiliae* isolates. Chromosome presence-absence variation (PAV) was analyzed by read mapping to the *Z. ardabiliae* Za100 PacBio genome assembly. Heatmap represents normalized mean coverage of reads mapped to each position of the genome assembly in bins of 10bp. Only unitigs larger than 100 kb are displayed and sorted by descending order of length. Darker colors represent regions of higher coverage as e.g. repetitive elements or duplications.

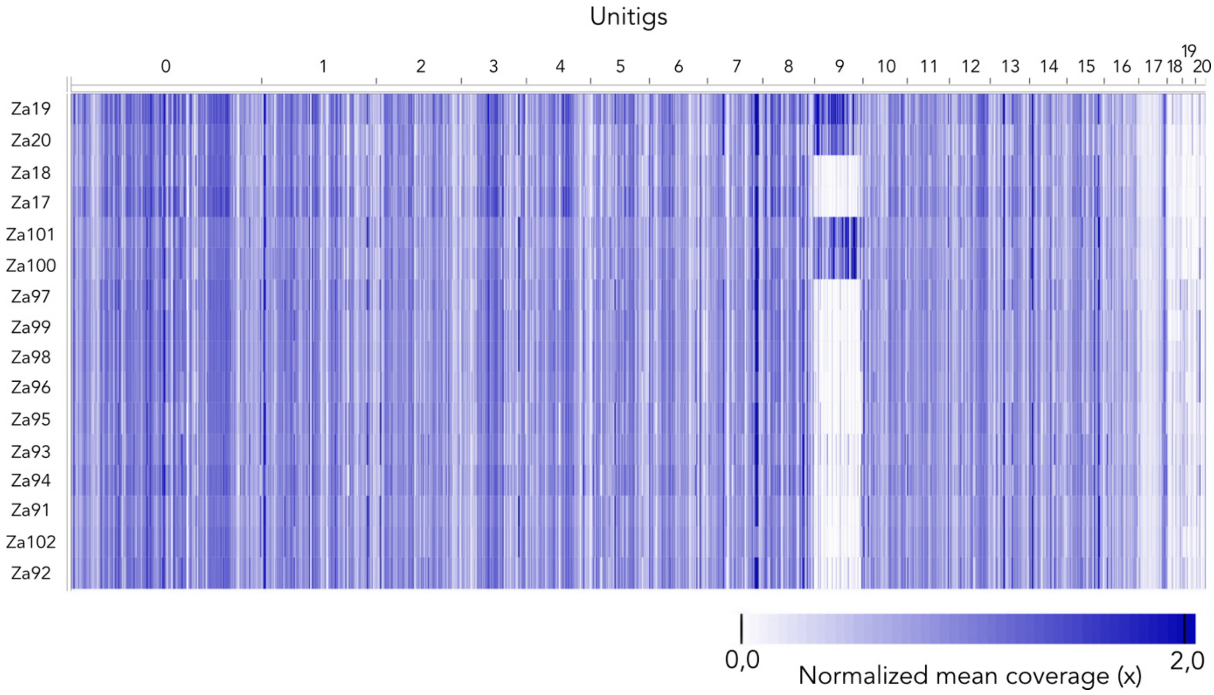

Figure S7. Read mapping to Zt469 genome confirms synteny and PAV of unitig 9 among *Z. ardabiliae* isolates. Chromosome presence-absence variation (PAV) was analyzed by read mapping to the *Z. tritici* Zt469 PacBio genome assembly and visualized using IGV v.2.8.2 (Robinson et al, 2011). Heatmap represents normalized mean coverage of reads mapped to each position of the genome assembly in bins of 10 bp. Except for unitig 9, only unitigs syntenic to the *Z. tritici* IPO323 reference genome are shown and sorted in descending order of length. Darker colors represent regions of higher coverage as e.g. repetitive elements or duplications.

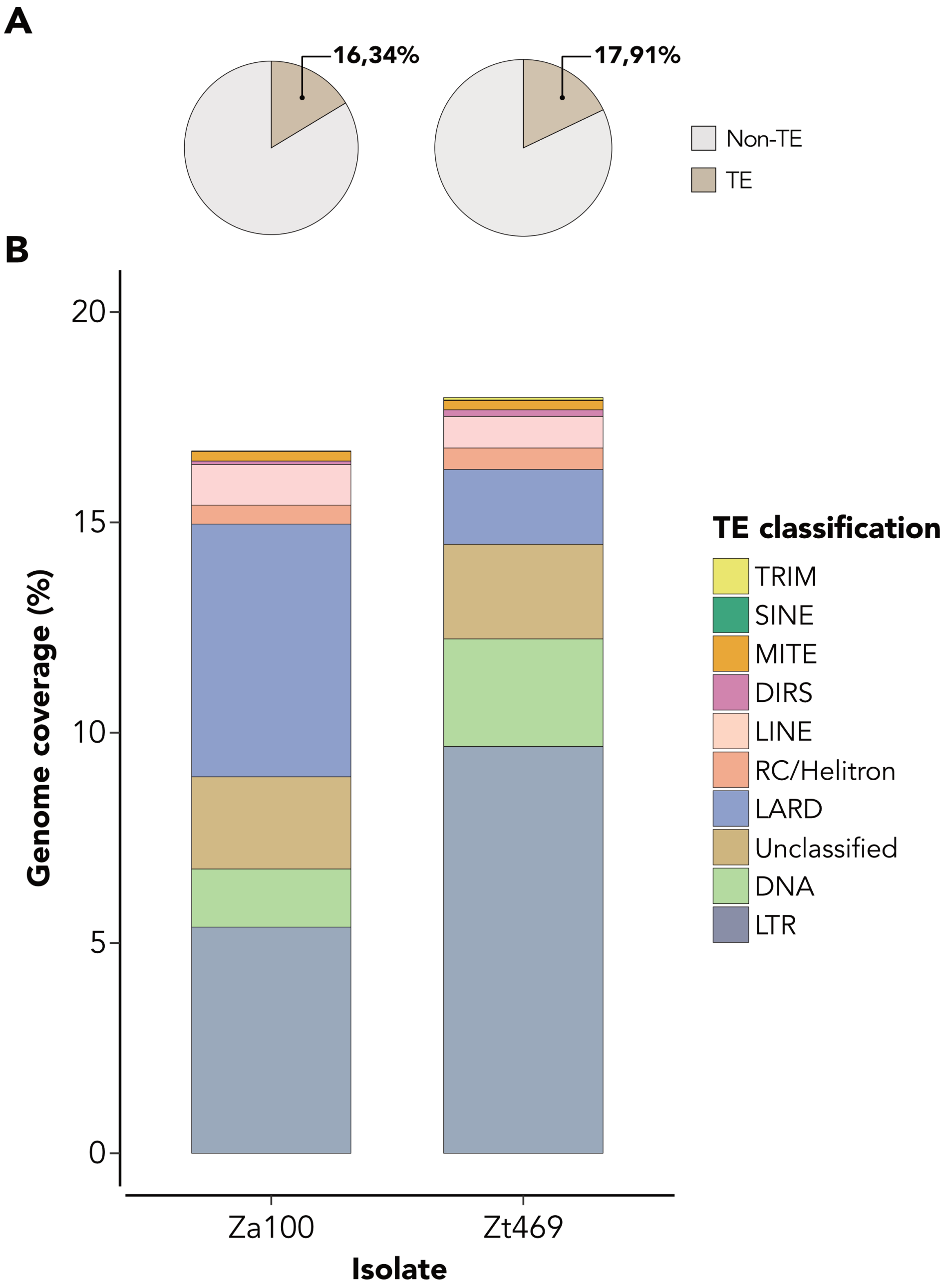

Figure S8. Transposable element (TE) content in Za100 and Zt469 genomes. **(A)** Total percentage of genome covered by TEs in Za100 (left) and Zt469 (right). **(B)** Stacked bar plot showing the TE content (%) per genome. Colors represent TE order coverage with retrotransposons (LTR, LINE, SINE, TRIM, LARD and DIRS) and DNA transposons (DNA, MITE, RC/Helitron). TEs that could not have been classified are indicated as “Unclassified”.

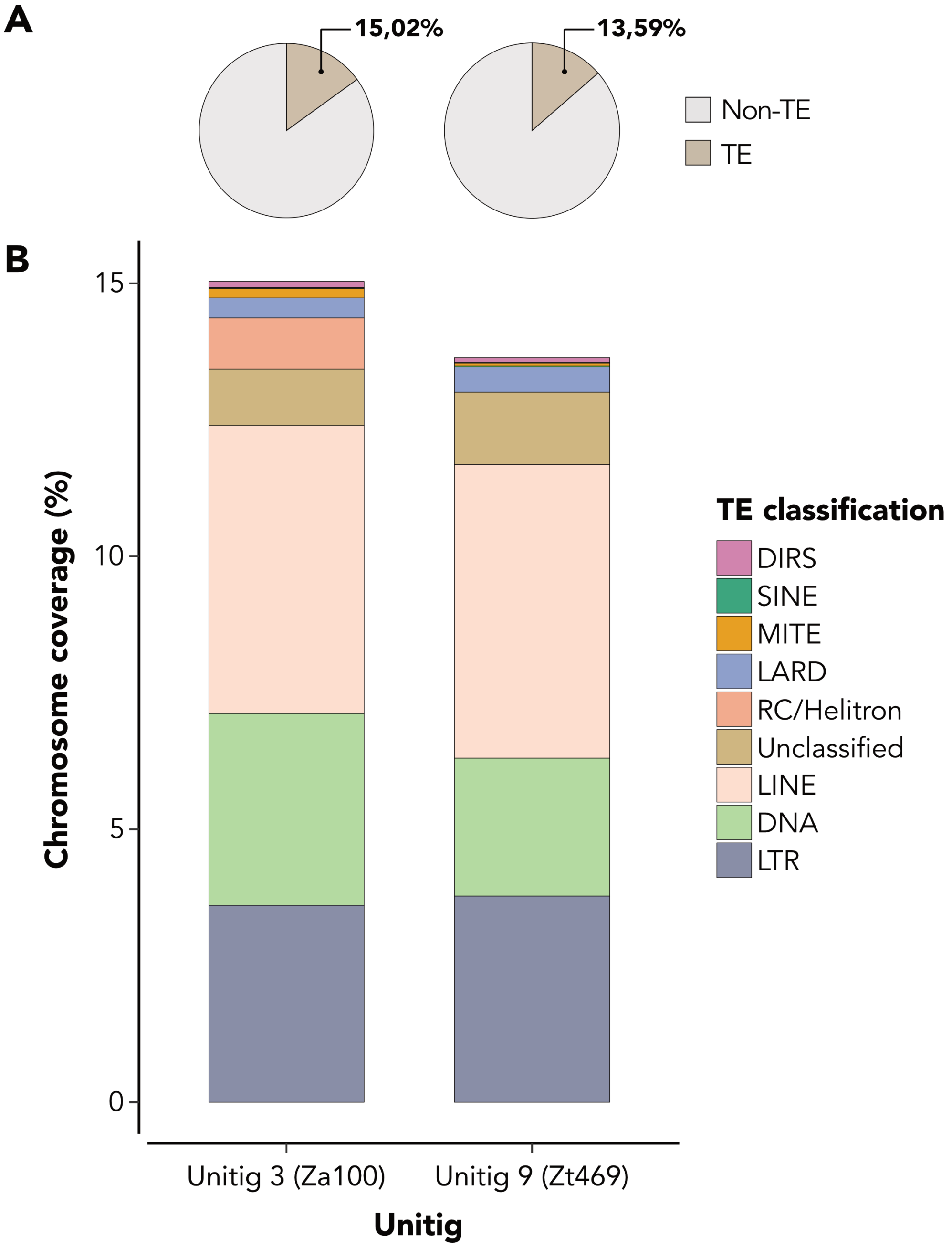

Figure S9. Transposable element (TE) content in the syntenic unitig 3 in Za100 and unitig 9 in Zt469. **(A)** Total percentage of unitig covered by TEs in unitig 3 (left) and unitig 9 (right). **(B)** Stacked bar plot showing the TE content (%) per unitig. Colors represent TE order coverage with retrotransposons (LTR, LINE, SINE, LARD and DIRS) and DNA transposons (DNA, MITE and RC/Helitron). TEs that could not have been classified are indicated as "Unclassified".

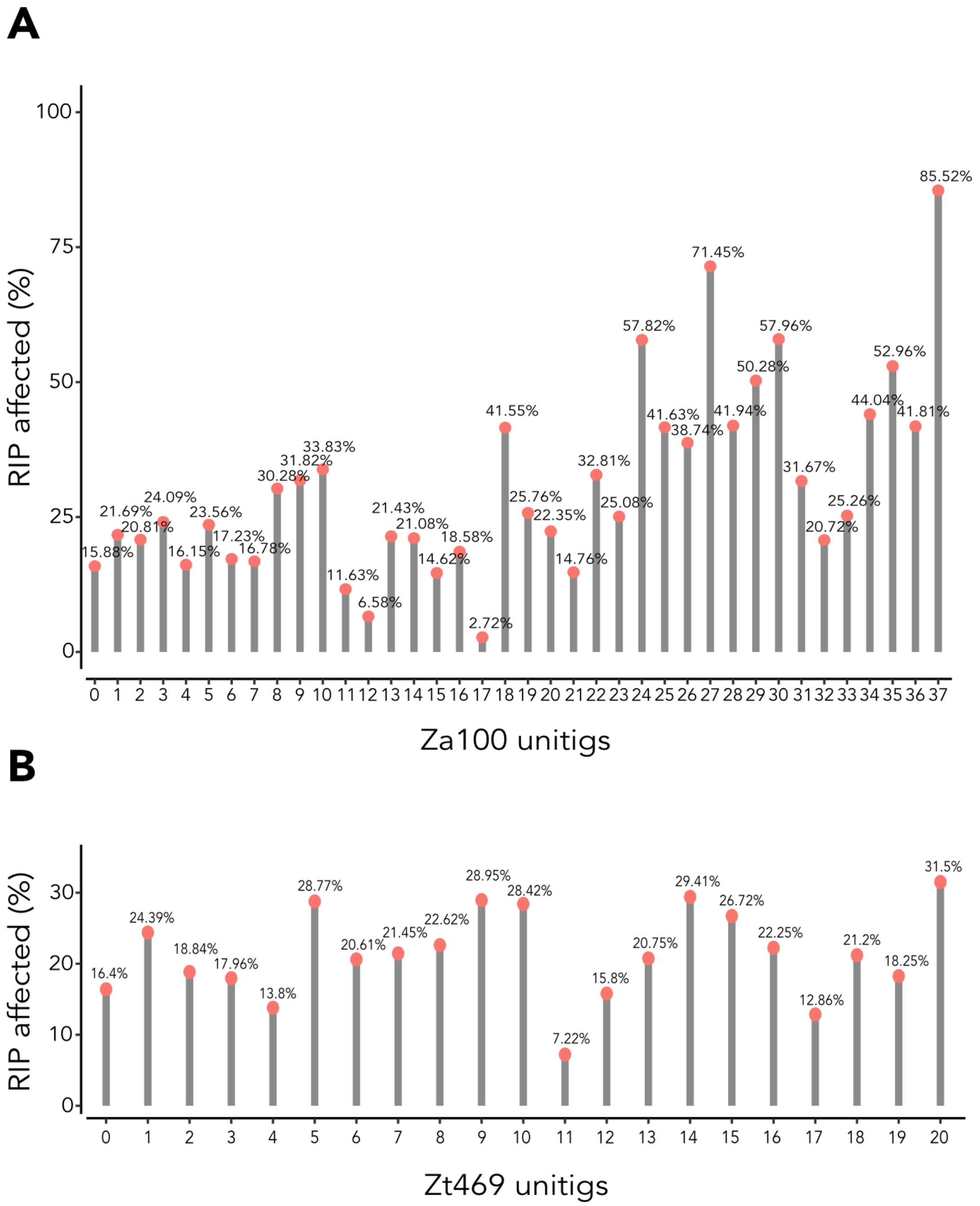

Figure S10. Unitig 3 and unitig 9 are differentially affected by RIP. Lolliplot plots showing the percentage of regions (1 kbp windows) per unitig affected by RIP in Za100 **(A)** and Zt469 **(B)** genomes**.** Unitigs are sorted by descending order of length. For Zt469 **(B)**, except for unitig 9, only unitigs syntenic to *Z. tritici* IPO323 reference genome are shown.

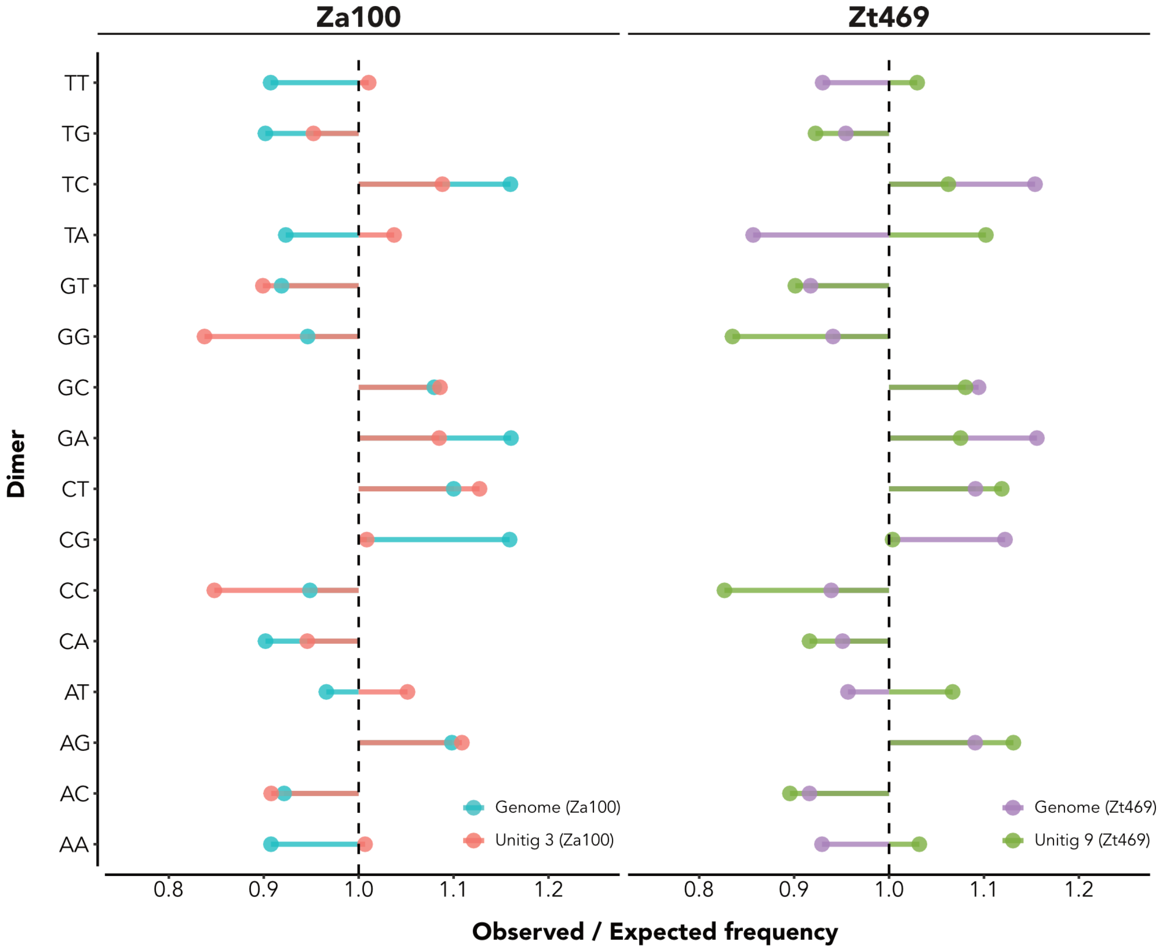

Figure S11. Za100 unitig 3 and Zt469 unitig 9 show higher frequency of "TA/AT" dimers than whole-genomes. Lollipop plots illustrate the dinucleotide (dimer) frequencies calculated for whole-genomes in Za100 (blue) and Zt469 (purple) as well as for unitig 3 in Za100 (red) and unitig 9 in Zt469 (green) specifically. Values report the observed versus expected dinucleotide frequencies. Expected frequencies are calculated on the (false) assumption that every dimer has equal frequencies (i.e. 0.0625).

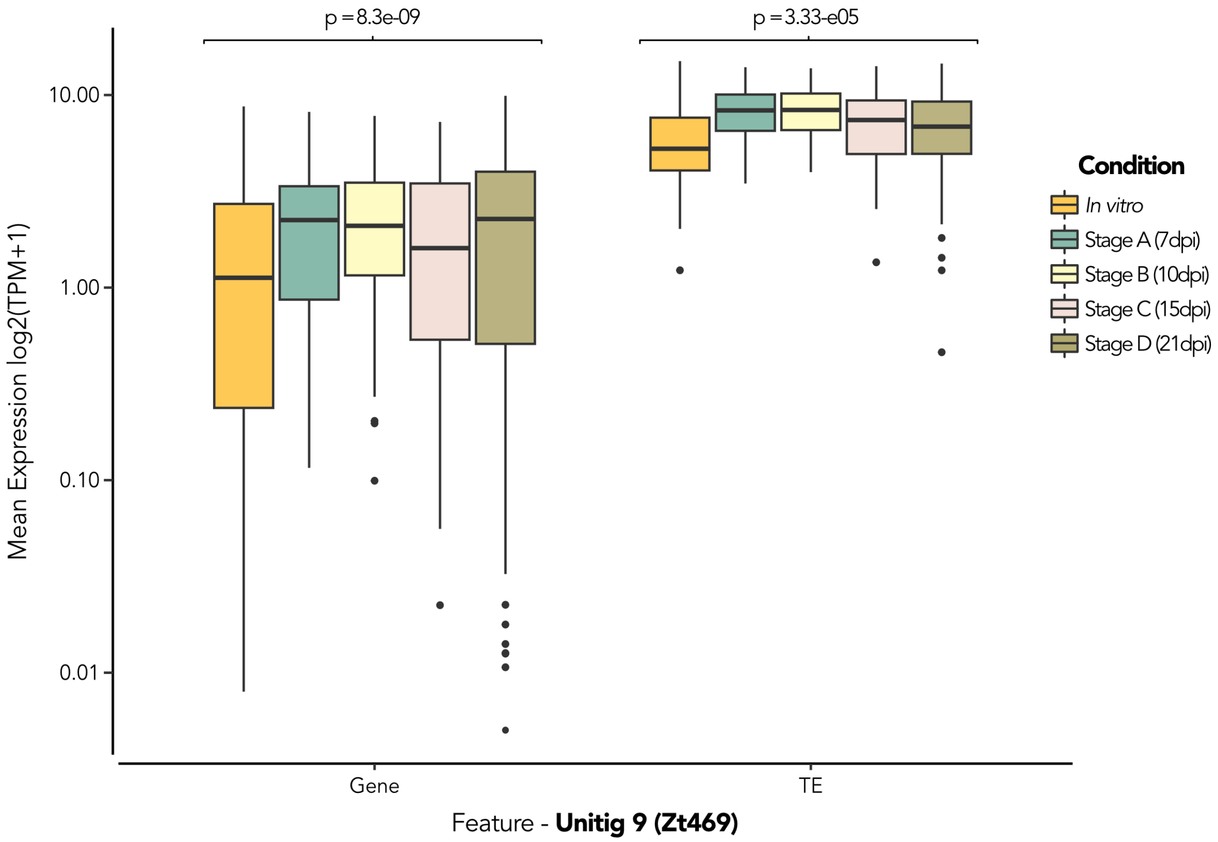

Figure S12. Genes and TEs show different levels of expression in Zt469 unitig 9 during *in vitro* growth and *in planta* infection. Boxplots representing the mean expression levels in log2(TPM+1) for genes and TEs in unitig 9 at different infection stages *in planta* and *in vitro*. P-values were calculated using Kruskall-wallis tests within each feature and between different conditions.

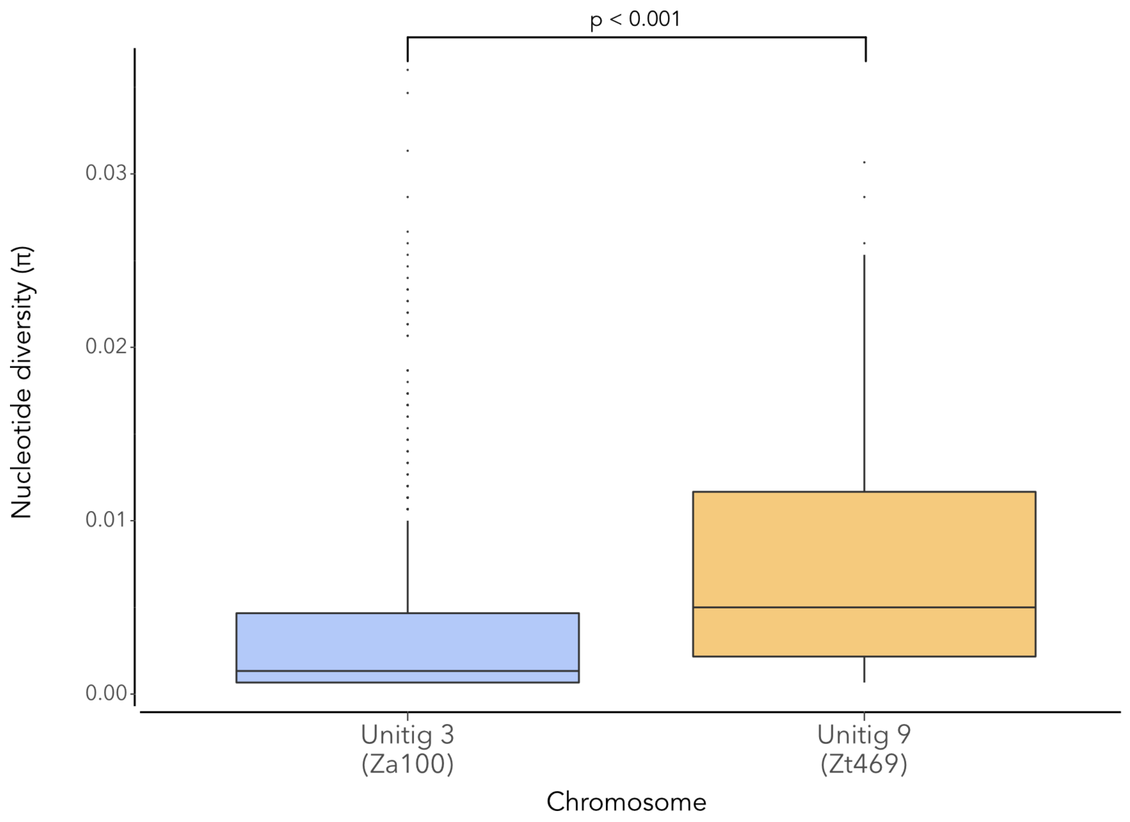

Figure S13. Unitig 9 shows higher nucleotide diversity. Boxplots represent the distribution of nucleotide diversity (π) values calculated per 1 kbp windows in unitig 3 (Za100) and unitig 9 (Zt469). P-value was calculated using Wilcoxon rank sum test.
